# Dual inhibition of CDK12 and CDK13 uncovers actionable vulnerabilities in patient-derived ovarian cancer organoids

**DOI:** 10.1186/s13046-023-02682-5

**Published:** 2023-05-18

**Authors:** Eleonora Cesari, Alessandra Ciucci, Marco Pieraccioli, Cinzia Caggiano, Camilla Nero, Davide Bonvissuto, Francesca Sillano, Marianna Buttarelli, Alessia Piermattei, Matteo Loverro, Floriana Camarda, Viviana Greco, Maria De Bonis, Angelo Minucci, Daniela Gallo, Andrea Urbani, Giuseppe Vizzielli, Giovanni Scambia, Claudio Sette

**Affiliations:** 1 Department of Neuroscience, Section of Human Anatomy, Catholic University of the Sacred Heart, 00168 Rome, Italy; 2 GSTeP Organoids Research Core Facility, IRCCS Fondazione Policlinico A. Gemelli, 00168 Rome, Italy; 3 Department of Woman and Child Health and Public Health, Catholic University of the Sacred Heart, 00168 Rome, Italy; 4 Department of Woman and Child Health and Public Health, Gynecologic Oncology Unit, Fondazione Policlinico Universitario A. Gemelli IRCCS, Largo Francesco Vito 1, 00168 Rome, Italy; 5 Unit of Translational Medicine for Woman and Child Health, IRCCS Fondazione Policlinico Universitario A. Gemelli, 00168 Rome, Italy; 6 Department of Diagnostic and Laboratory Medicine, Unity of Chemistry, Biochemistry and Clinical Molecular Biology, Fondazione Policlinico Universitario A. Gemelli IRCCS, Rome, Italy; 7 Department of Basic Biotechnological Sciences, Intensive Care and Perioperative Clinics Research, Catholic University of the Sacred Heart, Università Cattolica del Sacro Cuore, Rome, Italy; 8 Department of Medical Area (DAME), University of Udine, Udine, Italy; 9 Clinic of Obstetrics and Gynecology, “Santa Maria Della Misericordia” University Hospital, Azienda Sanitaria Universitaria Friuli Centrale, Udine, Italy

## Abstract

**Background:**

High grade serous ovarian cancer (HGSOC) is highly lethal, partly due to chemotherapy resistance and limited availability of targeted approaches. Cyclin dependent kinases 12 and 13 (CDK12/13) are promising therapeutic targets in human cancers, including HGSOC. Nevertheless, the effects of their inhibition in HGSOC and the potential synergy with other drugs are poorly known.

**Methods:**

We analyzed the effects of the CDK12/13 inhibitor THZ531 in HGSOC cells and patient-derived organoids (PDOs). RNA sequencing and quantitative PCR analyses were performed to identify the genome-wide effects of short-term CDK12/13 inhibition on the transcriptome of HGSOC cells. Viability assays with HGSOC cells and PDOs were performed to assess the efficacy of THZ531 as single agent or in combination with clinically relevant drugs.

**Results:**

The *CDK12* and *CDK13* genes are deregulated in HGSOC and their concomitant up-regulation with the oncogene *MYC* predicts poor prognosis. HGSOC cells and PDOs display high sensitivity to CDK12/13 inhibition, which synergizes with drugs in clinical use for HGSOC. Transcriptome analyses revealed cancer-relevant genes whose expression is repressed by dual CDK12/13 inhibition through impaired splicing. Combined treatment with THZ531 and inhibitors of pathways regulated by these cancer relevant genes (*EGFR*, *RPTOR*, *ATRIP*) exerted synergic effects on HGSOC PDO viability.

**Conclusions:**

CDK12 and CDK13 represent valuable therapeutic targets for HGSOC. We uncovered a wide spectrum of CDK12/13 targets as potential therapeutic vulnerabilities for HGSOC. Moreover, our study indicates that CDK12/13 inhibition enhances the efficacy of approved drugs that are already in use for HGSOC or other human cancers.

**Supplementary Information:**

The online version contains supplementary material available at 10.1186/s13046-023-02682-5.

## Background

High grade serous ovarian cancer (HGSOC) is the most aggressive subtype of ovarian cancer and a leading cause of gynecological cancer-related death in Western countries [1]. HGSOC is generally diagnosed at advanced stages and complete cytoreductive surgery, followed by platinum-based chemotherapy and maintenance therapy with inhibitors of poly (ADP-ribose) polymerase (PARPi) and/or vascular endothelial growth factor (VEGFi), represents the most curative treatment. Nevertheless, 70–80% of tumors relapse into more aggressive forms, leading to a 5-year survival rate < 50% [1, 2]. Thus, identification of more effective chemotherapeutic regimens and/or of therapies that directly target the molecular aberrations of the tumor are urgently needed to improve clinical management of HGSOC patients.

HGSOC is characterized by copy number changes and by mutations in genes that control the cellular response to DNA damage [3]. Most HGSOC (> 90%) harbor mutations in the *TP53* gene, which encodes the transcription factor p53 [4]. Upon DNA damage, p53 induces cell cycle arrest to allow repair of the lesion or, when the damage is too extensive, cell death. Cells lacking this checkpoint are prone to accumulate mutations upon DNA damage and to undergo neoplastic transformation [5]. Defective DNA repair via homologous recombination (HR) occurs in almost half of the cases and is frequently caused by inactivation of the breast cancer susceptibility genes *BRCA1* and *BRCA2* (overall up to 30%) [3, 4]. The presence of these mutations makes HGSOC particularly susceptible to DNA-damaging chemotherapeutic agents and explains the initial good response to treatments with carboplatin that is observed in most patients [1, 2]. Likewise, further impairment of the DNA damage response (DDR) by inhibition PARP enzymes, which flag the site of the DNA lesion to promote the assembly of the repair machinery [6], represents a recent advancement in the treatment of HGSOC patients [1, 2, 7]. However, not all patients respond equally to PARPi and resistance mechanisms often develop upon maintenance regimens with these drugs. Hence, identification of more efficacious combined treatments aimed at rapidly and completely eradicating the tumor cells before acquisition of resistance will likely improve life expectancy of HGSOC patients.

Mounting evidence points to the gene encoding the cyclin dependent kinase 12 (*CDK12*) as an important player in HGSOC. CDK12 is a transcriptional kinase, which phosphorylates serine 2 (Ser2) in the carboxyl-terminal domain (CTD) of the RNA polymerase II (RNAPII), thus promoting transcription elongation and RNA processing within the gene body [8–10]. While this activity is likely to occur genome-wide, inhibition or ablation of CDK12 function was shown to prevalently affect expression of DDR genes and to cause DNA repair defects [11], hence sensitizing different types of cancer cells to PARPi [12, 13]. *CDK12* appears to be frequently mutated in HGSOC patients [4] and the mutations were shown to impair the catalytic activity of the kinase and to cause defects in HR-mediated DNA repair [14, 15]. More recently, concomitant inhibition of CDK12 and its highly homologous kinase CDK13 was reported to exert strong anticancer activity in HGSOC cell and mouse models [16]. Transcriptome analyses revealed a widespread effect of CDK12/13 inhibition on HGSOC cells, confirming their role on the expression of DDR genes and the DNA repair pathway. Moreover, as reported in other cell types [17], the functions of CDK12 and CDK13 only partially overlap in HGSOC cells, and their concomitant inhibition elicited synergic effects with respect to single kinase ablation [16]. However, the mechanisms involved in the transcriptome reprogramming triggered by the CDK12/13 inhibitors were not investigated. In this regard, previous observations indicated that the effect of CDK12 inhibition on DDR gene expression was due to activation of cryptic polyadenylation (pA) sites in the introns of these genes, leading to premature termination of the encoded transcripts [18, 19]. Nevertheless, it is currently unknown if this mechanism also occurs in HGSOC cells and whether it is maintained in clinical-relevant models of the disease.

In this study, by using HGSOC cell lines and patient-derived organoids (PDOs) we confirmed the strong anticancer activity of CDK12/13 inhibition. High-throughput RNA sequencing analyses highlighted intron retention (IR) and consequent premature transcript termination as key mechanisms of gene inactivation in HGSOC cells. In addition to DDR genes, our analysis identified other actionable targets of CDK12/13, including the Epidermal Growth Factor Receptor (*EGFR*) and the Regulatory Associated Protein of MTOR Complex 1 (*RPTOR*) genes. Combined treatment with a CDK12/13 inhibitor increased the sensitivity of HGSOC cells and PDOs to inhibitors of EGFR (Lapatinib) and of the mechanistic target of rapamycin complex 1 (MTORC1; RAD001 or Everolimus), as well as to Paclitaxel, PARPi and ATR/CHK1 inhibitors, suggesting the clinical relevance of this mechanism of action for anticancer treatment.

## Methods

### Cell cultures

The OVCAR3 and OV-90 cell lines were obtained from the American Type Culture Collection (ATCC, Milan, Italy). OVCAR3 cells were cultured in RPMI 1640 (Roswell Park Memorial Institute Medium) supplemented with 10% fetal bovine serum (FBS) and OV-90 in 1:1 mixture of MCDB 105 medium (containing a final concentration of 1.5 g/L sodium bicarbonate) and Medium 199 (containing a final concentration of 2.2 g/L sodium bicarbonate) supplemented with 15% FBS. All contained 1% MEM (Minimum Essential Medium) Non-Essential Amino Acid, 1 mM glutamine and 1% penicillin and streptomycin. The OV.GEM9 and OV.GEM11 cell lines were established by primary serous ovarian carcinoma specimens as previously described [20], and were maintained in complete DMEM/F12 (1:1) supplemented with 10% heat inactivated FBS, 2 mM glutamine and 2 mM kanamycin (Life Technologies, CA, USA). All cells were grown in a humidified incubator at 37 °C and 5% CO_2_.

### Patient-derived organoids

Tumour biopsies were collected from patients treated at “Fondazione Policlinico Universitario A. Gemelli IRCCS” (FPG), Rome, Italy, from May 2020 to October 2022. The protocol was approved by the Institutional Review Board (Protocol ID: 3046) and conducted in accordance with the Helsinki Declaration. All patients enrolled gave their written informed consent for participation. Relevant clinical data were collected and managed using REDCap electronic data capture tools [21] at FPG (https://redcap-irccs.policlinicogemelli.it). Ovarian cancer tissues were placed in 60 mm Petri dishes containing AdDF +  +  + culture medium (Advanced DMEM/F12 containing 1 × Glutamax, 10 mM HEPES and antibiotics). Part of the tissue was fixed in formalin for histopathological and immunohistochemistry analysis, part was stored at − 80 °C for DNA/RNA isolation. The remaining part was minced by surgical blades into small fragments for organoids generation and digested in 10 ml AdDF +  +  + supplemented with 5 µM RHO/ROCK pathway inhibitor (Y-27632, Tocris) containing 2 mg/ml Collagenase IV (Termofisher) on an orbital shaker at 37 °C for 1 h. The cell suspension was then applied to a MACS SmartStrainer (100 μm), placed on a 50 mL tube and washed with 10 ml of AdDF +  +  + culture medium and centrifugated at 290* g*. The pellet was incubated with 1 ml red blood cell lysis buffer for 5 min at room temperature to eliminate erythrocytes, followed by washing with culture medium and pelleting at 290* g.* Cells were embedded in undiluted (100%) Cultrex growth factor reduced BME type 2 (Trevigen) on ice and 40 µl drops of BME cell suspension were allowed to solidify to a pre-warmed 24 well suspension culture plates (Greiner). The plate was placed at 37 °C for 30 min to allow the Matrigel to polymerize before being overlaid with 500 µl of a growth factor cocktail medium and incubated at 37 °C in humidified air containing 5% CO2. Organoid culture medium was AdDF +  +  + culture medium supplemented with EGF (100 ng/ml), A83-01 (0.5 μM), B27 (1X) and N2 (1X) supplement, Y-27632 (9 µM), Nicotinamide (1 mM) and Primocin. Medium was changed every 3–4 d and organoids were passaged every 1–4 weeks by incubation with Cultrex Organoid Harvesting Solution for 45 min at 4 °C to digest the BME. PDOs were then mechanically dissociated by pipetting or by enzymatic digestion with Triple Express (Gibco) for 5–15 min at 37 °C, if needed. After rinsing in PBS, organoid fragments were resuspended in cold BME and reseeded as above at suitable ratio (1:1 to 1:3). Genomic analysis of PDOs and original tumors were carried out using the TruSight Oncology (TSO) 500 by Next Generation Sequencing technology (Illumina Inc.).

### Histological analysis and immunohistochemistry

Five µm sections of paraffin-embedded tissues and PDOs were stained with haematoxylin and eosin (H&E) and immunohistochemistry staining was performed with appropriate primary monoclonal rabbit anti-human antibodies: WT1 (clone 6F-H2, Roche Diagnostics, Indianapolis, IN), PAX8 (PAX8-EP331), p53 (clone Bp53-11 Roche), anti-CINtec ® Histology Kit (p16) (Roche) using the UltraView Universal DAB Detection Kit on the ULTRA instrument (Ventana) BenchMark. For cytokeratin 7 (CK7; clone OV-TL 12/30 DAKO/AGILENT) and CK20 (Clone Ks20.8 DAKO/AGILENT) staining was performed on the Bond III automated immunostainer (Leica Microsystems, Bannockburn, IL). Appropriate positive and negative controls were included. The selected antibodies are well-established in the diagnostic routine laboratory, signals were clearly visible and captured by ordinary light microscope (Zeiss AxioPhot Microscope). Scores were performed by an expert pathologist.

### TCGA Somatic mutation and clinical data analysis

Somatic mutation, gene expression and clinical data of Ovarian Serous Cystadenocarcinoma patients (TCGA PanCancer Atlas, *n* = 398) were retrieved from cBioPortal portal (https://www.cbioportal.org/). For oncoplot somatic mutation data were analyzed using R package ComplexHeatmap to identify and summarize somatic mutations in the R version 4.1.1. For gene expression level of *CDK12* and *CDK13* respect to *MYC* expression, patients were divided into two groups according to the median of *MYC* gene expression (high above, low below the median value). Z-scores value of *CDK12* and *CDK13* was calculated in each sample and significance of differential expression between the two groups was calculated by Mann–Whitney test. Overall survival was used for Kaplan–Meier analysis. Patient’s high- and low-expression groups were compared using median cut-off modus and *p* value calculated with Gehan-Breslow-Wilcoxon test (GraphPad Prism 8).

### Viability assays

For cell lines, cells were seeded in 96-well plate overnight at a density of 1.5/3 × 10^3^ per well, and then treated with drugs at indicated concentrations alone or in combination. After incubation, 20μL of CellTiter 96® AQueous One Solution Cell Proliferation Assay (MTS Assay, Promega) was added into each well for 1 h. The OD (optical density) of each sample was detected at 490 nm using a microplate reader (Biorad). For the analysis of cytotoxicity, cells were incubated with the Incucyte® Cytotox Dye, which fluorescently labels the nuclei of dead cells when membrane integrity is lost. Dying cells were identified and quantified over time by the appearance of green/NIR labeled nuclei. For all experiments, each treatment was performed in biological triplicates.

For PDOs, after the dissociation by pipetting or with Triple Express, organoids fragments are resuspended in 2% BME/growth medium and seeded in 100 µl volume on BME pre-coated 96-well plates. After 24 h, the cells were treated with the indicated drugs for 5 days and viability was determined by CellTiter-Glo® Luminescent Cell Viability Assay (Promega) using a microplate reader (Spark, Tecan).

Viability was determined relative to the control cells exposed to vehicle and analyzed using GraphPad Prism 9 (GraphPad Software, San Diego, CA, USA). Drug dose–response curves were visualized using linear regression analysis (setting: log(inhibitor) versus normalized response). Half-maximal inhibitory concentration (IC50) values were determined from fitting curves.

For synergic interaction analyses, cells and PDOs were exposed to individual drug at various concentrations alone or in combination with suboptimal doses of the other compound. Drug combination effects were estimated based on the combination index (CI) values calculated using the Compusyn software (Biosoft, Ferguson, MO, USA), as described [22]. A CI < 1 indicates synergism.

### RNA extraction and PCR analysis

Total RNA was extracted using RNA Mini Kit (Geneaid) and 1 μg of RNA was retro-transcribed with oligo-dT oligonucleotides, using M-MLV reverse transcriptase (Promega). 20 ng of cDNA was used as template for PCR (GoTaq, Promega) and reactions were analyzed on agarose or acrylamide gels. Quantitative real-time PCRs (qPCR) were performed using LightCycler 480 SYBR Green I Master and the LightCycler 480 System (Roche), according to the manufacturer’s instructions. Control reactions omitting M-MLV reverse transcriptase were also carried out. PCR oligonucleotides are listed in Table S1.

### RNA-Seq and bioinformatics analyses

Total RNA was extracted and DNase-treated using the miRNEasy extraction kit (QIAGEN) from OVCAR3 cells treated with 200 nM THZ531 or DMSO for 6 h. PolyA plus RNA-seq libraries were constructed and sequenced using a 150 bp paired-end format on an Illumina NovaSeq6000 (GSE222493). RNA-seq data analysis was performed previously described [23–25]. Briefly, sequencing, data quality, reads repartition (e.g., for potential ribosomal contamination), inner distance size estimation, genebody coverage, strand-specificity of library were performed were performed using FastQC, Picard-Tools, Samtools and Rseqc. Reads were mapped using STARv2.4.0 on the human hg38 genome assembly and read count was performed using featureCount from SubRead. Analysis at gene expression level was performed using Human FAST DB v2021_3 annotations [25] and DESeq2. Results were considered statistically significant for fold-change ≥ 1.5 and *p*-value ≤ 0.05. Splicing analyses were performed taking as previously described [24, 25] and results were considered statistically significant for *P*-values ≤ 0.05 and fold-changes ≥ 1.5 for “PATTERN” analysis and *P*-values ≤ 0.01 and fold-changes ≥ 2.0 for “EXON” analysis. For gene set enrichment analysis (GSEA) all genes were ranked based on the log2 expression and then subjected to GSEA analysis using the cancer hallmark gene sets from MSigDB. The enrichment score (ES) was calculated for each functional set, which reflects the degree to which a gene set is overrepresented at the top or bottom of the ranked list of genes. Normalized enrichment score (NES) was calculated based on 1000 permutations. Expression levels, gene length, ratio between introns and exons lengths and intron size for genes regulated at the gene expression (GE), RNA processing (RP), both gene expression and RNA processing (GE/RP) and unregulated genes were evaluated and represented using custom R script. The relative position of THZ531-regulated introns in the transcription unit was evaluated by calculating the ratio between the position of the regulated introns versus the total number of introns/exons for each gene. To visualize their distribution a density curve was drawn using ‘‘density’’ function. Analysis for enriched GO biological processes and KEGG Pathway was performed using Enrichr platform (https://maayanlab.cloud/Enrichr/), using the upregulated IR and alternative last exon (ALE) events.

### Fluorescence-activated cell sorting (FACS) analysis

For cell cycle and proliferation analyses OVCAR3 cells were treated with 100 nM and 200 nM THZ531 or DMSO for 24 h and pulse-labelled with 30 µM BrdU (Sigma-Aldrich) for 1 h. Cells were collected, fixed overnight with a solution of 70% ethanol in PBS (vol/vol) at 4 °C and, after ice-cold PBS washes, stained with 50 µl of anti-BrdU antibody (0.02 µg/µl) diluted in PBS supplemented with 1% BSA and 0.5% Tween-20, for 1 h at RT. After washes in PBS, cells were incubated with 50 µl of Alexa-488-conjugated secondary antibody (0.02 µg/µl), for 45 min at RT and treated with 10 µg/ml RNAse A (Merck, Sigma-Aldrich) in presence of propidium iodide (20 µg/ml) (Merck, Sigma-Aldrich). A total of 20,000 events were counted with FACS-Calibur flow cytometer (Becton Dickinson) using Cell Quest software (Becton Dickinson) and analysed using FlowJo software.

### Western blot analysis

Whole cell proteins were obtained by lysing the cells with RIPA lysis buffer in the presence of proteases and phosphatase inhibitors. Equal amounts of protein were separated by SDS polyacrylamide gel electrophoresis, blotted to PVDF (Polyvinylidene fluoride), and transferred using the Trans-Blot Turbo Transfer System (Bio-Rad) with 25 V, 1.0 A, for 30 min. After blocking in 5% non-fat milk, membranes were probed with the following primary antibodies: 1:1000 anti-ATRIP, 1:1000 anti-EGFR, 1:1000 anti–phospho 4EBP1, 1:1000 anti–4eBP1 (all from Cell Signaling Technology), 1:500 anti-PAX-8 (Proteintech Group Inc), 1:1000 anti-RNAPII phospo-Ser2 (Millipore), 1:1000 anti-RNAPII (Bethyl) and 1:1000 anti-HSP90 (Santa Cruz Biotechnology) at 4 °C, overnight. After incubation with secondary horseradish peroxidase-conjugated antibody, proteins were visualized by the enhanced chemiluminescence system (Bio-Rad or Amersham Biosciences, Buckinghamshire, UK) using a ChemiDoc imaging system (UVITEC, Cambridge).

### Statistical analyses

The prognostic effect of various parameters on clinical outcome, as overall survival (OS), was tested by plotting survival curves according to Kaplan–Meier method and median survival was calculated. Cell proliferation data were analyzed by one-way analysis of variance (ANOVA), using Tukey's multiple comparison test to determine if significant differences existed between groups. qPCR data were analysed by the two-tailed unpaired Student’s t-test. All data are reported as mean ± SEM (n ≥ 3) and statistical analyses were performed using the GraphPad Prism9 Software.

## Results

### The *CDK12* and *CDK13* genes are deregulated in ovarian cancer and their expression is associated with poor prognosis in MYC-overexpressing patients

**Fig. 1:**
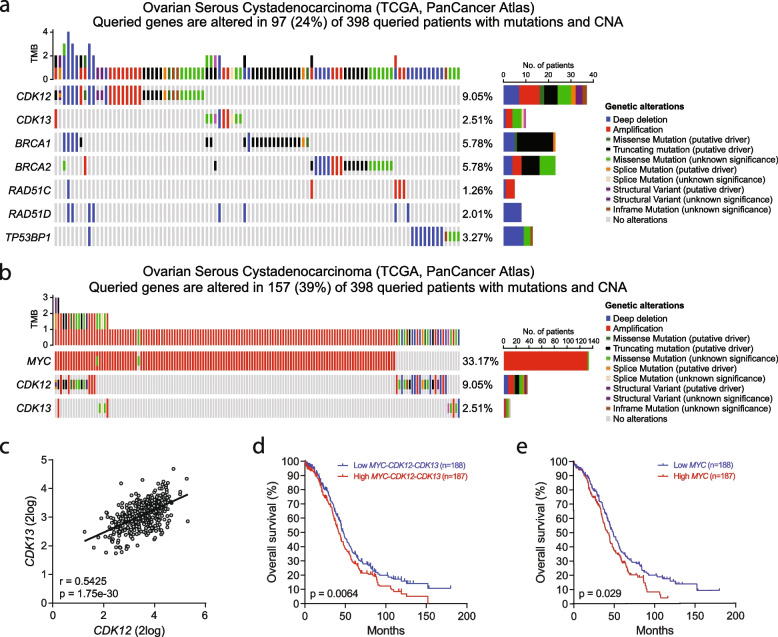
CDK12 and CDK13 alterations in Ovarian Serous Cystadenocarcinoma. **a**, **b** Oncoplots showing the distribution of mutations in *CDK12*, *CDK13*, *MYC* and selected DDR genes in ovarian serous cystadenocarcinoma patients analyzed in the Pancancer Atlas project of TCGA. The Tumor Mutational Burden (TMB) plot shows the frequency of mutations for each tumor sample. The lower plots show the type of mutation fpr each indicated gene in each tumor sample. The orizontal bar graphs (right side) report the number of samples mutated. **c,** Pearson’s correlation plot of the expression levels of *CDK12* and *CDK13* transcripts, data from 375 ovarian serous cystadenocarcinoma patients deposited in the Pancancer Atlas project of TCGA. Pearson’s correlation coefficient (r) and *P*-value are reported. **d**, **e** Kaplan–Meier curves comparing the overall survival of patients described above with concomitant high (red) or low (blue) expression of *MYC, CDK12, CDK13* transcripts (**d**) or with high (red) or low (blue) expression of *MYC* transcript (**e**)

Analysis of data from The Cancer Genome Atlas (TCGA) indicated that the frequency of alterations in the *CDK12* (9%) and *CDK13* (2.5%) genes was similar (*CDK13*) or higher (*CDK12*) with respect to other DDR genes among ovarian cancer patients (*n* = 398; Fig. 1a). Moreover, they were almost mutually exclusive, as only one patient harbored a concomitant *CDK12* truncating mutation and *CDK13* amplification (Fig. 1a). Approximately half of the alterations in *CDK12* were missense, splice or truncating mutations, while the remaining ones were equally distributed between deletion and amplification of the gene (Fig. 1a). A similar distribution was observed also for *CDK13*, albeit this gene was more frequently amplified than deleted in ovarian cancer patients (Fig. 1a). With the exception of one patient harboring a *CDK12* deletion, all patients that carry deep deletions of either *CDK12* or *CDK13* also carry mutations in at least one other DDR gene (Fig. 1a), suggesting that these tumors have a higher mutational burden. Nevertheless, more than 80% of ovarian cancer patients do not present alterations in the *CDK12* and *CDK13* genes (Fig. S1a).

Recent evidence indicated that ovarian cancer cells depend on the oncogenic transcription factor MYC for their growth and survival [26]. The *MYC* gene is frequently amplified in ovarian cancer (> 30% of patients; Fig. 1b). Moreover, HGSOC cell lines overexpressing *MYC* were shown to depend on the activity of transcription-associated CDKs, including CDK12/13 [26]. Thus, we also asked whether there is a correlation between *MYC* amplification and the mutational status of the *CDK12/13* genes in ovarian cancer. Analysis of the same cohort of patients form the PanCancer project showed that *CDK12* and *CDK13* deep deletions are mutually exclusive with *MYC* amplification in ovarian cancer (Fig. 1b). *CDK12* was significantly more expressed in patients segregated for high *MYC* expression (*MYC*^high^; Fig. S1b), while *CDK13* showed a similar, yet not significant, trend (Fig. S1c). We also found a significant positive correlation (*r* = 0.54; *p* = 1.75^e−30^) between *CDK12* and *CDK13* expression in HGSOC patients (Fig. 1c). Furthermore, while high expression of *CDK12* and *CDK13* was not associated with survival (Fig. S1d,e), their concomitant high expression with *MYC* is a significant prognostic marker of poor outcome with higher statistical significance than high *MYC* alone (*p* = 0.0064 versus *p* = 0.029; Fig. 1f,e). These analyses support the important role played by CDK12 and CDK13 in ovarian cancer.

### Dual CDK12/13 inhibition strongly impairs survival of HGSOC primary cell models

Since > 80% of patients express wild type CDK12 and CDK13, we set out to evaluate the impact of their concomitant inhibition in HGSOC cells. Treatment of OVCAR3 cells with THZ531, a specific CDK12/13 dual inhibitor [27], impaired cell viability in a dose-dependent manner, with an almost complete effect at 200 nM (Fig. S2a). Similar results were observed with SR4835 (Fig. S2a), another dual CDK12/13 inhibitor with a different chemical structure [28]. CDK12/13 inhibition also impaired viability of another HGSOC cell line (OV-90; Fig. S2b) and of two previously characterized HGSOC primary cell lines (OV.GEM9 and OV.GEM11) [20] that displayed intrinsic resistance to carboplatin (IC50 > 100 µM; Fig. S2c,d).

As previously reported in other cell lines [17, 19] treatment of OVCAR3 cells with THZ531 repressed Ser2 phosphorylation in RNAPII (Fig. S2e). Upon CDK12/13 inhibition, OVCAR3 cells arrested in the S (S BrdU^−^) and G2/M phase of the cycle (Fig. S2f,g). This block was likely caused by unrepaired DNA breaks, as indicated by accumulation of G2/M cells that are positive for the DNA damage marker γH2AX (Fig. S2h). Accordingly, THZ531 treatment increased the sub-G1 population and cytotoxicity in OVCAR3 cells (Fig. S2i,j). These data confirm the key role played by CDK12/13 in HGSOC cells and suggest their potential efficacy as therapeutic targets for this cancer type.

**Fig. 2:**
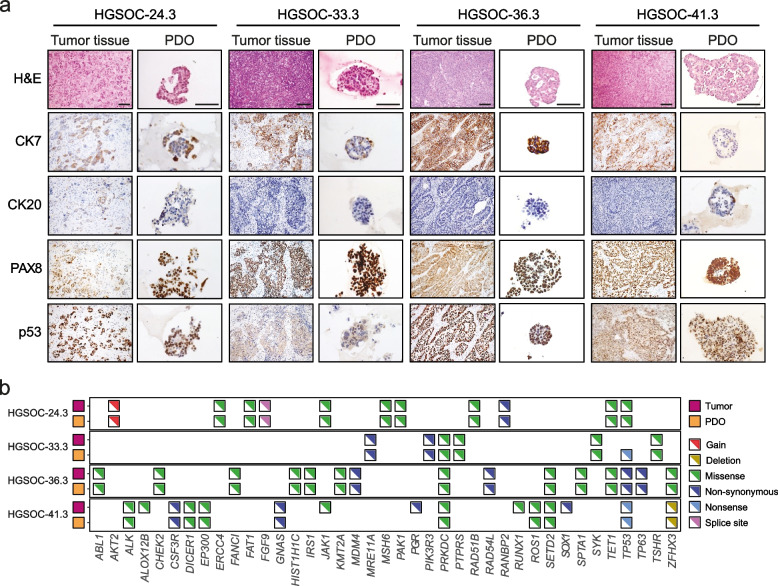
PDOs retained histological and genomic features of the original tumor. **a** Histological comparison of representative HGSOC PDOs and their parental tumor tissue. The panel shows hematoxylin eosin (H&E) staining, cytokeratin 7 (CK7), CK20, PAX8 and p53 immunostaining in HGSOC tumor tissue (magnification 20 ×) and corresponding PDOs (magnification 40 ×). Scale bar, 100 μm. **b** Somatic mutations and amplifications/deletions in relevant genes of ovarian cancer. For each sample, tumor/organoid pairs are displayed and indicated by color coding (pink, tumors; orange, organoids)

PDOs are emerging as powerful tools in molecular oncology, as they can be exploited to bridge the gap between *ex-vivo* studies and the clinical response of patients to treatments [29, 30]. PDOs have been also established from HGSOC and were shown to faithfully recapitulate the features of the tumor of origin [31–33]. Thus, to investigate the biological activity of CDK12/13 inhibition in a clinically relevant context, we set out to develop PDOs from HGSOC patients undergoing primary debulking surgery (PDS) or diagnostic laparoscopy (Table S2), who were then followed for response to treatments. By using biopsies comprising variable percentages of tumor cells, we developed six PDO lines that could be propagated for more than 5 passages (Table S2). All PDOs maintained the phenotypic features of the tumor of origin and expressed the expected HGSOC markers, such as p53, the Wilm’s tumor protein (WT1) and cytokeratin 7 (CK7; Fig. 2a; Table S2). Likewise, genomic analysis of four PDO lines mostly confirmed the same mutation pattern in PDOs and their tumor of origin (Fig. 2b). An exception was the nonsense (truncating) mutation in TP53 found only in PDO33.3. However, since this mutation is predicted to cause delocalization of p53 in the cytoplasm and this pattern was observed in both PDO33.3 and its original tumor, it is likely that non tumoral cells in the original tissue masked the mutation [32]. We also detected five mutations (*ALOX2B*, *JAK1*, *PGR*, *RUNX1*, *SOX1*) in the HGSOC41.1 tumor sample but not in the corresponding PDO (Fig. 2b). No mutations in *CDK12* and *CDK13* genes were detected in these HGSOC tumor specimens or PDOs. Thus, with few exceptions, these results confirm that the HGSOC PDOs faithfully recapitulate the pathophysiological and genetic phenotype of the original tumor.

**Fig. 3:**
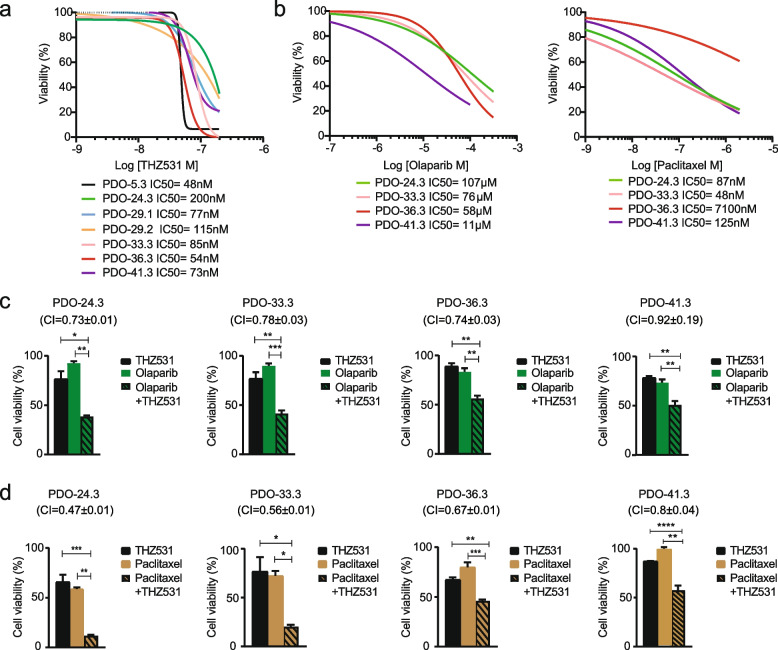
CDK12/13 inhibition impairs viability of HGSOC PDOs and synergizes with Olaparib and Paclitaxel. **a**, **b** Cytotoxic effects of THZ531 (**a**), Olaparib and Paclitaxel (**b**) on the indicated PDO lines. Cells were exposed to various concentrations of the drugs for 5 days and viability was evaluated by Cell Titer Glo 3D assay. Half-maximal inhibitory concentration (IC50) values were determined from fitting curves using GraphPad Prism 9.0. All results represent the mean ± SEM of technical triplicates. **c**,**d**, Synergic effects of THZ531 and Olaparib (**c**) or Paclitaxel (**d**) on viability of the indicated PDO lines. Organoids were exposed for 5 days to combined treatments with suboptimal doses of THZ531 (30 nM and 50 nM), Olaparib (1 and 10 µM) and Paclitaxel (2 and 20 nM) (*P < 0.05, **P < 0.01, ***P < 0.001). CI values < 1, which suggest synergism, was calculated for drug combinations relative to the individual drugs and are indicated above the graphs. All results are expressed as the mean ± SEM derived from technical triplicates

As observed in the clinical response of most HGSOC patients [1, 2], viability assays indicated that all the established PDOs are sensitive to carboplatin, as they display an IC_50_ below the maximal concentration achievable in patient’s plasma (concentration steady state/maximum concentration Css/Cmax = 53.7 µM) (Fig. S2k) [34]. Furthermore, treatment of the PDO lines with increasing doses of THZ531 indicated their high sensitivity to the drug, with an IC_50_ ≤ 200 nM for all cultures (Fig. 3a), which was accompanied by reduced phosphorylation of Ser2 in RNAPII (Fig. S2l).

Next, we asked whether CDK12/13 inhibition could sensitize tumors that are wild type for *BRCA1/2* to PARPi, as previously shown for some HGSOC cell lines [16]. Genetic analysis of primary biopsies indicated that HGSOC-24.3, HGSOC-33.3, HGSOC-36.3 and HGSOC-41.3 tumors and corresponding PDOs were wild type for *BRCA1/2* (Fig. 2b; Table S2). Accordingly, all these PDO lines were insensitive to Olaparib, with IC_50_ above (range 11–107 µM) the in vivo Css/Cmax (3.4 µM) of the drug (Fig. 3b, left panel). However, combined treatment with suboptimal doses of THZ531 (IC_30_, 30-50 nM) and Olaparib (1–10 µM) synergically reduced the viability of all four PDO lines (combination index, CI < 1) (Fig. 3c). Interestingly, these PDO lines showed a wide range of sensitivity to treatment with Paclitaxel (IC_50_ = 48 nM -7.1 µM; Fig. 3b, right panel), a first-line chemotherapeutic drug for HGSOC that is administered with carboplatin [1]. THZ531 significantly enhanced the effect of Paclitaxel in all PDOs tested (Fig. 3d), including PDO-36.3 that was intrinsically resistant to the drug (IC_50_ = 7.1 µM; Css/Cmax = 4.3 µM). The synergic effect of the THZ531/Paclitaxel combination was strong for PDO-24.3 (CI = 0.47), PDO-33.3 (CI = 0.56) and PDO-36.3 (CI = 0.67) with respect to the PDO-41.3 (CI = 0.8), suggesting a patient-specific response to the combined treatment.

These results indicate that primary human HGSOC models strongly depend on CDK12/13 function for survival, that CDK12/13 inhibition overcomes innate resistance to PARPi and that tumors may respond differently to combined regimens with Paclitaxel and THZ531.

### CDK12/13 inhibition elicits a widespread impact on the HGSOC transcriptome

**Fig. 4:**
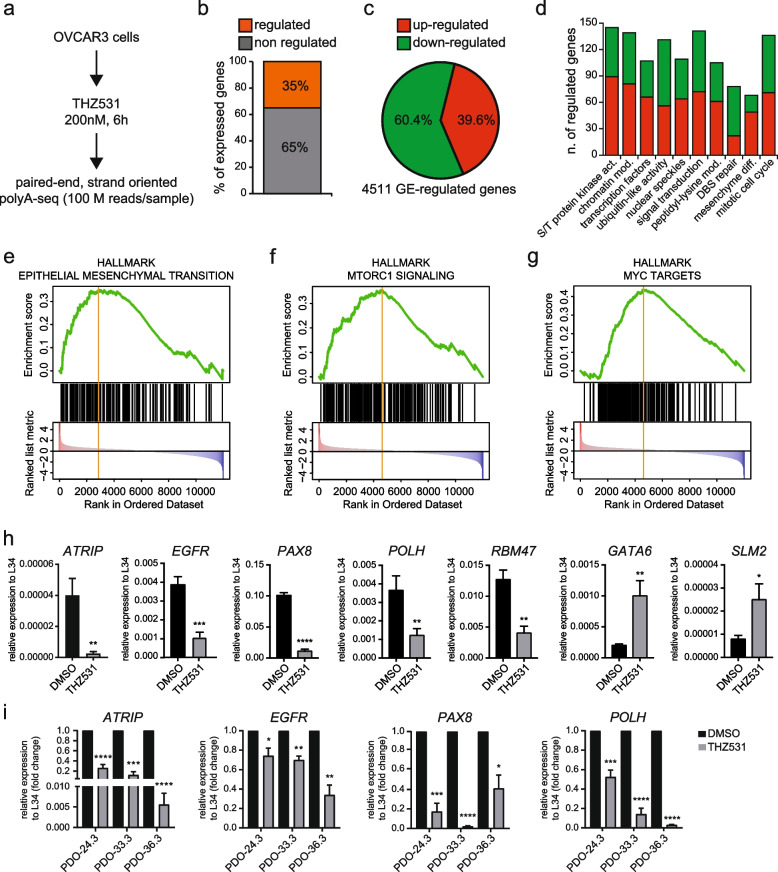
THZ531 widely affects the transcriptome of HGSOC cells. **a** Schematic representation of the experimental workflow used for generating the RNA-seq datasets of OVCAR3 cells treated with either DMSO or THZ531 (200 nM) for 6 h. **b** Bar graphs representing the percentage of genes regulated (orange bar) or not (grey bar) by treatment with THZ531. **c**, Pie chart showing percentages of genes whose expression is either up- (red) or down-regulated (green) by treatment with THZ531. **d** Bar graph showing the functional categories (KEGG Pathway) most significantly affected by treatment of OVCAR3 cells with THZ531. Height of each bar represents the number of differentially expressed gene; up-regulated genes are shown in red while down-regulated genes are shown in green. **e–g** Gene Set Enrichment analysis (GSEA) of epithelial mesenchymal transition (**e**) mTORC signaling (**f**) and *MYC* targets (**g**) signatures in OVCAR3 cells treated with THZ531. Bars represent individual genes in a ranked data set list. **h**, Bar graphs show the results of qPCR analyses of the *ATRIP, EGFR,PAX8, POLH, RBM47, GATA6* and *SLM2* transcripts relative to L34 in OVCAR3 cells treated or not with THZ531 (mean ± SEM; *n* = 3; *P < 0.0001). **i** Bar graphs show the results of qPCR analyses of the *ATRIP, EGFR* and *PAX8* transcripts relative to L34 in OVCAR3 cells treated or not with THZ531 (mean ± SEM; *n* = 3; *P < 0.05,**P < 0.01, ***P < 0.001, ****P < 0.0001)

CDK12/13-dependent phosphorylation of the RNAPII is required for efficient transcription elongation [8, 10]. Furthermore, pharmacologic inhibition of these kinases was shown to widely affect the transcriptome of multiple cell lines [11, 17–19, 27, 28], including HGSOC cells [16]. To gain more insight into the gene expression program governed by CDK12/13 in HGSOC, we carried out a genome-wide transcriptome analysis of OVCAR3 cells treated with THZ531 for 6 h (Fig. 4a). High coverage (> 100 millions reads/sample) RNA sequencing (RNA-seq) analyses indicated that control (DMSO) and treated (THZ531) samples (*n* = 3) clustered separately (Fig. S3a). Bioinformatic analysis using the FAST-DB database as reference [25] revealed that short-term treatment with THZ531 altered the expression (fold change ≥ 1.5; *p* ≤ 0.05) of 35% of the genes expressed in OVCAR3 cells (Fig. 4b; Table S3). While most genes were down-regulated (60,4%), as expected from the inhibition of transcriptional kinases, a substantial amount of them was also up-regulated (Fig. 4c). In line with the known role of CDK12 in the regulation of HR [11] and with the G2/M arrest observed in our experiments, double strand break (DSB) repair and mitotic cell cycle were among the functional categories enriched in THZ531-regulated genes (Fig. 4d). In addition, this analysis uncovered other cancer-relevant functional categories, such as signal transduction proteins, transcription factors, splicing speckles and chromatin modifiers (Fig. 4d). Furthermore, Gene Set Enrichment Analysis (GSEA) highlighted the effect of THZ531 on pathways of crucial relevance for many human cancers [35], including MYC target genes, epithelial-to mesenchymal transition (EMT) and mTORC1 signaling (Fig. 4e-g). To validate the RNA-seq analysis, we selected seven representative genes on the basis of their relevance for HGSOC (*PAX8*, *EGFR*) and other cancers (*GATA6*, *RBM47*, *KHDRBS3*), or for their involvement in the DDR pathway (*ATRIP*, *POLH*). Analysis by quantitative real time PCR (qPCR) using a different set of OVCAR3 cells confirmed our bioinformatics predictions (Fig. 4h).

Next, to assess whether the transcriptome dysregulation caused by CDK12/13 inhibition in OVCAR3 cells was recapitulated in primary HGSOC models, we selected three PDO lines displaying different sensitivity to THZ531 (range 54–200 nM; Fig. 3a). Analysis of *ATRIP*, *EGFR*, *PAX8* and *POLH* expression showed that treatment with THZ531 caused downregulation of all genes in HGSOC PDOs (Fig. 4i), with the extent being generally correlated with sensitivity to the drug (Fig. 3a). Thus, CDK12/13 inhibition exerts a widespread and reproducible effect on the HGSOC transcriptome, which leads to dysregulation of multiple oncogenic pathways and processes.

### Inhibition of CDK12/13 activity impairs RNA processing in HGSOC cells

**Fig. 5:**
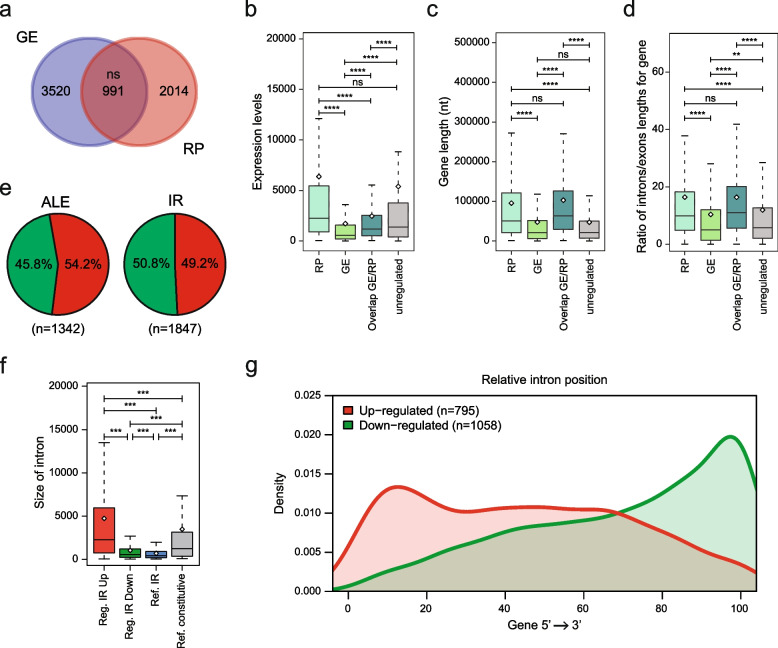
CDK12/13 inhibition dysregulate RNA processing events in HGSOC cells. **a** Venn diagram showing the overlap between genes regulated at the gene expression (GE) and RNA processing (RP) level. Statistical significance was evaluated by the hypergeometric test. **b-d** Box plots representing distribution of the expression level (**B**), gene length (**C**) and intron/exon lengths (**d**) among genes regulated at the gene expression (GE), RNA processing (RP), both gene expression and RNA processing (GE/RP) and unregulated genes. Whiskers indicate 1.5 interquartile range. *P*-values above the graph indicate significant difference between the mean value of the indicated groups(**P < 0.01, ****P < 0.0001, ns = not significant; t-test). **e** Pie charts showing numbers and percentages of up- (red) and down-regulated (green) alternative last exon (ALE, left) and intron retention (IR, right) events in THZ531-treated cells. **f**, Box plots showing the distribution of the size of introns up- and down-regulated, non-regulated intron retention events annotated in the FAST-DB database (Ref. IR), constitutively spliced introns (Ref. constitutive). **g** Metagene plot showing the position distribution of up- (red line, *n* = 795) and down-regulated (green line, *n* = 1058) introns within the gene body (0–100%; 5’-to-3’ orientation)

Ser2 phosphorylation in the CTD is required for the transition from transcription initiation to elongation and to maintain RNAPII processivity within the gene body [36]. CDK12/13 are thought to promote coupling between the elongating RNAPII and RNA processing (RP) factors [8–10], as their inhibition caused IR, premature cleavage and polyadenylation of pre-mRNAs [17–19, 37]. Accordingly, CDK12/13 inhibition widely affected RP (fold change ≥ 1.5; *p* ≤ 0.05) of thousands of transcripts in OVCAR3 cells (Fig. 5a, Fig. S3b; Table S3). Less than one third of the splicing-regulated genes were also affected at the overall gene expression (GE) level (Fig. 5a), suggesting that CDK12/13 inhibition results in different outcome on different types of target genes.

Next, we set out to investigate the structural features that are associated with regulation at GE and RP level, or both. Genes regulated by THZ531 at GE (*n* = 3520) and GE/RP level (*n* = 991) are expressed in OVCAR3 cells at significantly lower level than unregulated (*n* = 6048) and RP-regulated (*n* = 2014) genes (Fig. 5b). RP- and GE/RP-regulated genes are longer than unregulated and GE-regulated genes (Fig. 5c) and display a higher intron/exon length ratio between (Fig. 5d). Thus, in addition to regulating overall transcription, inhibition of CDK12/13 affects RP in genes that are characterized by long introns. This observation is consistent with previous studies indicating that inhibition of CDK12 causes selection of alternative last exons (ALEs) and/or intronic cleavage and polyadenylation in genes characterized by large introns [17–19, 37]. Indeed, we detected thousands of ALE and IR events regulated by short-term treatment with THZ531 (Fig. 5e). Direct validation of two of these ALE events (*MMS22L* and *CSTF3*) confirmed the reliability of the bioinformatics analysis (Fig. S3c). Notably, up-regulated introns are characterized by a particularly large size with respect to all other categories (Fig. 5f) and are specifically distributed in the 5' proximal region of the coding unit of the THZ531-target genes (Fig. 5g). Since up-regulation of ALE and IR generally leads to alteration of the open reading frame by causing premature cleavage and polyadenylation of the transcript [38], with direct functional consequences, we further focused on these RP events.

### CDK12/13 inhibition causes intron retention and premature termination of cancer-relevant transcripts in HGSOC organoids

**Fig. 6:**
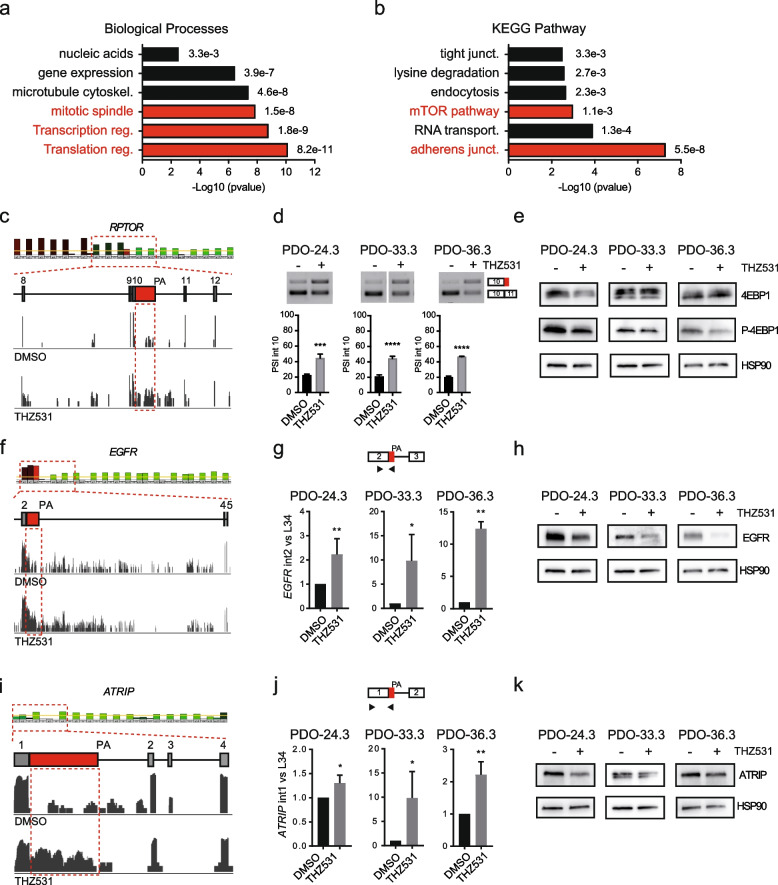
Inhibition of CDK12/13 impairs splicing and expression of cancer-relevant genes in HGSOC. **a** Gene Ontology analyses of the biological processes significantly affected by up-regulated IR and ALE events in HGSOC cells. **b** KEGG pathway analysis of the genes significantly affected by up-regulated IR and ALE events in HGSOC cells. **c**, **f**, **i**, Profiles of the RNA-seq reads of the IR events in *PAX8* (**c**), *EGFR* (**f**), *ATRIP* (**i**) genes in OVCAR3 cells treated with DMSO (upper graph) or THZ531 (lower graph). Sequence reads (vertical gray lines), exons (grey boxes), and introns (horizontal lines and red boxes) are shown. **d**, **g**, **j**, Schematic representation for each event analysed (top panels): black arrows in the scheme indicate primers used for the PCR analysis. Bar graphs (bottom panels) show the results of qPCR analyses for the expression of the retained intron (red boxes) relative to L34 (Data represent mean ± SD of three biological replicates; ∗ P ≤ 0.05, **P ≤ 0.01; t-test). **e**, **h**, **k** Western blot analyses of the expression of PAX8 (**e**), EGFR (**h**), ATRIP (**k**) proteins in PDO cell extracts. HSP90 was included as a loading control

Most human introns contain cryptic pA sites, which can be selected to generate ALEs comprising intronic sequences in frame with the upstream exon. IR favors the recognition and usage of such intronic suboptimal pA sites by the cleavage and polyadenylation complex [9, 38]. Thus, both ALE and IR impair the production of the full-length protein encoded by the gene and likely alter its function(s). Annotation of the genes affected by increased ALE (*n* = 728) and IR (*n* = 794) highlighted biological processes and pathways of direct relevance for human cancers, such as the regulation of RNA transcription, processing and translation, mitotic spindle organization, cell adhesion, apoptosis and DNA damage repair (Fig. 6a,b). Among the CDK12/13-targets involved in transcription, PAX8 is a well-established diagnostic marker of ovarian cancer [39]. Recent evidence suggested that *PAX8* is regulated at transcriptional level by CDK12/13 [40]. Accordingly, this gene is strongly repressed upon treatment of HGSOC cells and PDOs with THZ531 (Fig. 4h,i). However, inspection of the RNA-seq data indicated that THZ531 treatment caused a large increase in read coverage in the proximal part of intron 2, up to a cryptic pA site (AAUAAA) located 1771 nucleotides downstream of the 5ʹ splice site (Fig. S4a). Increased retention of the proximal portion of intron 2 was validated by qPCR analysis in THZ531-treated OVCAR3 cells and PDO-36.3 (Fig. S4b,c), and likely accounts for the observed reduction in full-length PAX8 mRNA and protein expression (Fig. S4b,c). THZ531 also caused up-regulation of a proximal ALE within intron 10 of *RPTOR* (Fig. 6c). The RPTOR protein positively regulates the activity of the mTORC1 complex, a central regulator of mRNA translation and protein synthesis [41]. We confirmed usage of the ALE in intron 10 in both PDOs and OVCAR3 cells (Fig. 6d; Fig. S4d), leading to down-regulation of more distal exons (Fig. 6c). Selection of this ALE in RPTOR was correlated with reduced phosphorylation of the mTORC1 substrate 4E-BP1 (Fig. 6e; Fig. S4d), indicating its negative impact on the pathway.

IR and ALE events were also enriched in genes associated with the adherens junction pathway (Fig. 6b), including the gene encoding for the epidermal growth factor receptor (*EGFR*). EGFR promotes proliferation in epidermal cells and its activity allows survival of HGSOC cells in nonadherent conditions and induces their peritoneal spread, suggesting that it represents an actionable target for this disease [42]. As for *PAX8*, inhibition of CDK12/13 induced retention of the proximal portion of *EGFR* intron 2 in OVCAR3 cells (Fig. 6f), likely due to premature cleavage and polyadenylation. Analysis by qPCR confirmed the up-regulation of intron 2 upon THZ531 treatment in both PDOs (Fig. 6g) and OVCAR3 cells (Fig. S4e). This effect was functionally relevant, as it correlated with reduced EGFR protein expression (Fig. 6h, Fig. S4e). Thus, inhibition of EGFR expression may contribute to the reduced proliferation of THZ531-treated PDOs and OVCAR3 cells.

Another interesting THZ531 target is *ATRIP*, which encodes a protein required for the recruitment of the DDR kinase ATR to DNA lesions [43]. Also in this case, THZ531 caused the up-regulation of a proximal intron in *ATRIP* (intron 1), downregulation of the downstream region (Fig. 6i,j; Fig. S4f) and reduced protein expression (Fig. 6k; Fig. S4f). Since ATRIP promotes ATR-dependent signaling in the DDR, particularly in the replication fork stress pathway [43], its reduction may account for the observed accumulation of unrepaired DNA lesions in the G2 phase of HGSOC cells treated with THZ531 (Fig. S2h).

Noteworthy, analysis of independent datasets (PRJNA789153) from OVCAR8 cells treated with THZ531 or another CDK12/13 inhibitor (ZSQ836) [16] confirmed the downregulation of these four genes (Fig. S5a). Moreover, analysis of the read coverage highlighted impairment of intron splicing in these genes (Fig.S5b-e), as we detected in OVCAR3 cells. Collectively, these results points to defective splicing of introns as a main effect of CDK12/13 inhibitors, which contributes to their anti-oncogenic effects by functionally inactivating genes with direct relevance for HGSOC.

### CDK12/13 inhibition uncovers actionable vulnerabilities in HGSOC organoids

Pharmacologic inhibition of the EGFR, mTORC1 and ATR pathways represent therapeutic options in several cancer types. Since CDK12/13 inhibition impairs the expression or activity of proteins in these pathways, we asked whether treatment with THZ531 enhances the sensitivity of HGSOC cells to inhibitors that are being evaluated in clinical trials or already approved. The IC_50_ of Lapatinib (EGFR inhibitor), RAD001 (Everolimus, mTORC1 inhibitor), Ceralasertib (ATR inhibitor) and AZD7762, an inhibitor of the ATR downstream effector kinase CHK1, was first estimated in OVCAR3 cells (Fig. S6a-d). Combined treatment with suboptimal doses of THZ531, exerted a synergic effect (CI < 1) with all these clinically approved drugs (Fig. S6f-i). As THZ531 concomitantly reduces EGFR expression and mTORC1 signaling, we also asked whether its strong effect on cell viability could be reproduced by combining inhibitors of these two pathways. Indeed, treatment with suboptimal doses of RAD001 and Lapatinib or Trametinib, a clinically approved inhibitor of the EGFR effector kinases MEK1 and 2 [44], elicited a synergic effect on OVCAR3 cell viability (Fig. S6e,j,k). Likewise, we found that combined treatment with Ceralasertib and Olaparib synergically affected cell viability (Fig. S6l).

**Fig. 7:**
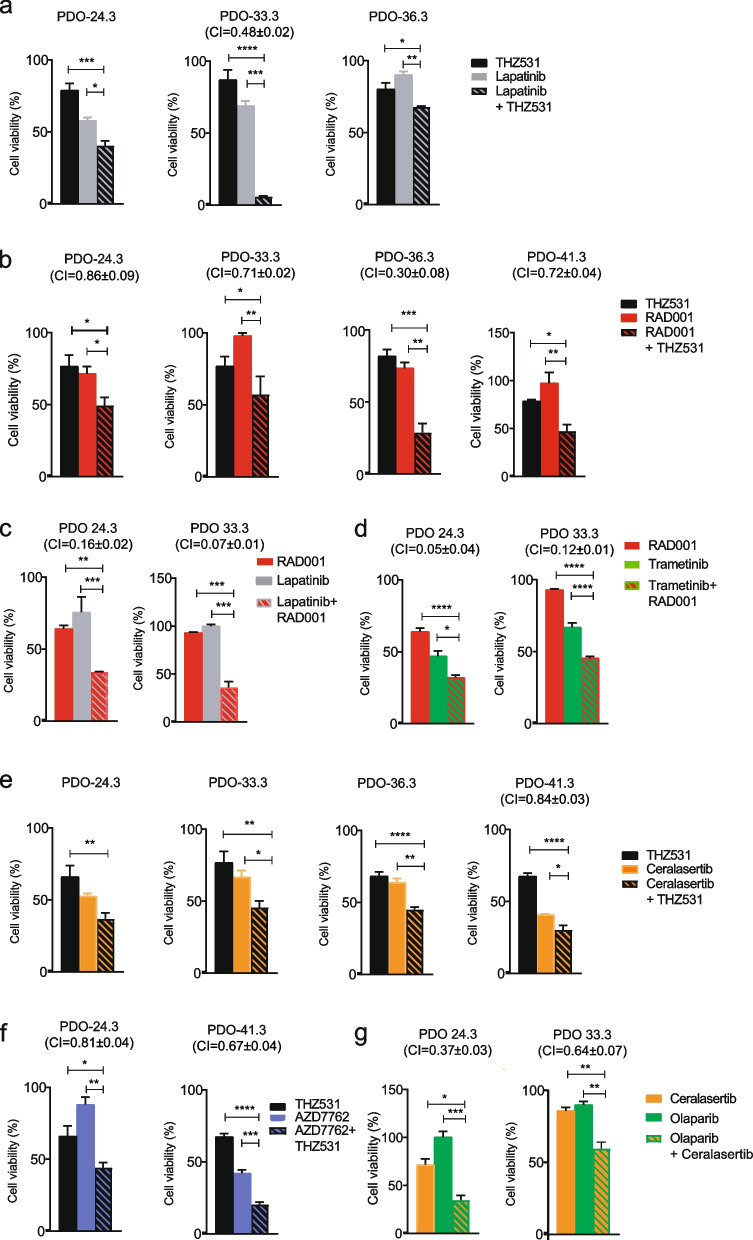
THZ531 synergizes with clinically approved inhibitors of the EGFR, mTORC1 and ATR signalling pathways. PDO lines were exposed for 5 days to combined treatments with suboptimal doses of THZ531 (30 nM and 50 nM), (**a,b,e,f**) and Lapatinib (0.5 and 1 µM) (**a**), RAD001 (10 and 100 nM) (**b**), Celarasertib (1 and 2 µM) (**e**) and AZD7762 (100 nM) (**f**) or with suboptimal doses of RAD001 (10 nM) and Lapatinib (0.1 µM) (**c**) or RAD001 (10 nM) and Trametinib (1 and 5 µM) (**d**). Moreover, PDO lines were exposed with suboptimal doses of Celarasertib (1 µM) and Olaparib (0.1 and 1 µM) (**g**). Cell viability was assessed by CellTiter-Glo 3D Cell Viability Assay. Statistically significant differences are indicated by the *P*-values (*P < 0.05, **P < 0.01, ***P < 0.001). The calculated CI values for drug combination relative to the individual drugs are presented above the graphs. CI values less than 1 suggest synergism. All results are expressed as the mean ± SEM derived from technical triplicates

Next, we tested whether the synergism between these drugs was maintained in primary HGSOC models. The IC_50_ for Lapatinib, Ceralasertib and AZD7762 in PDOs was within a similar range of that observed in OVCAR3 cells, whereas PDOs were relatively unaffected by RAD001 as single agent (Fig. S7a-d). Conversely, PDOs were more sensitive to Trametinib than OVCAR3 cells (Fig. S7e). Combined treatment with THZ531 and Lapatinib exerted a stronger effect on survival of all PDO lines tested with respect to either agent alone. However, the extent of the effect was variable, being mild in PDO-24.3 and PDO-36.3 and synergic (CI = 0.48) in PDO-33.3 (Fig. 7a). Noteworthy, PDO-33.3 displayed the lowest sensitivity to Lapatinib as single agent (IC50 = 1.3µM; Fig. S7a), suggesting that concomitant CDK12/13 inhibition may overcome an intrinsic resistance of this specific tumor to EGFR inhibition. On the other hand, combined treatment with THZ531 and RAD001 exerted a synergic effect in all PDO lines (Fig. 7b). Furthermore, the combination between RAD001 and Lapatinib or Trametinib exerted a strong synergic effect also on PDO viability (CI < 0.2; Fig. 7c,d), indicating that these clinically approved drugs may results useful in HGSOC.

A tumor-specific effect was also observed in combined treatments of THZ531 and ATR or CHK1 inhibitors (Fig. 7e,f; Fig. S7f). While a stronger effect of combined treatments was observed in most cases, Ceralasertib synergized with THZ531 only in PDO-41.3 (CI = 0.84), whereas AZD7762 plus THZ531 synergically reduced survival of PDO-24.3 (CI = 0.81) and PDO-41.3 (CI = 0.67). Moreover, Ceralasertib synergized with Olaparib in HGSOC PDOs (Fig. 7g). Thus, inhibition of CDK12/13 enhances the sensitivity of HGSOC cells to chemotherapeutic agents already in use for this disease (i.e. Paclitaxel and Olaparib) and uncover new potential vulnerabilities to drugs (i.e. Lapatinib, Trametinib, RAD001, Ceralasertib) that are clinically approved or in trial for other cancer types.

## Discussion

CDK12 and CDK13 are emerging as suitable targets in human cancers [9, 10], including HGSOC [16]. Mutational data from TCGA indicate that > 80% of patients harbor wild type or amplified *CDK12/13* genes, and mutation of these genes is mutually exclusive with *MYC* amplification. Thus, CDK12/13 likely play an oncogenic role in a large fraction of ovarian cancer patients. These kinases promote the elongation rate of the RNAPII within gene units, an activity that is particularly relevant for the expression of DDR genes [11, 17–19]. Herein, we confirmed the high sensitivity of HGSOC cells to CDK12/13 inhibition. Moreover, THZ531 strongly impaired viability also of HGSOC cell lines that were resistant to carboplatin, suggesting that it could be a valuable second-line drug for relapsing tumors. Transcriptome analyses uncovered a wide range of genes whose expression is affected by these kinases, which extend beyond the well-known DDR genes. CDK12/13 inhibition impairs expression of several cancer-relevant genes by causing premature termination of their transcripts and reduction of the corresponding proteins. As exemplified by *EGFR* and *RPTOR*, these new CDK12/13 target genes function in pathways that are actionable in HGSOC, as clinically approved inhibitors have shown efficacy in other cancer types. Thus, our work highlights a spectrum of potential vulnerabilities in HGSOC that could be further explored through the development of target-specific drugs and/or chemical inhibitors.

A novelty of our investigation is represented by the employment of HGSOC organoid models. Since HGSOC is heterogeneous and the response to treatments varies among patients diagnosed with apparently similar diseases [1, 2], the use of established cell lines in most previous studies on CDK12/13 function might represent a limit. Herein, we developed PDOs from tumor biopsies of six patients who underwent PDS and were then followed for response to treatments. PDOs were validated by analysis of HGSOC biomarkers, which confirmed that they mostly maintained the phenotype of the original tumor. We also detected few mutations exclusively in the HGSOC41.1 tumor sample but not in the corresponding PDO. This discrepancy could be related to the cellular heterogeneity of the tumor. Indeed, recent work suggested that organoids can also be generated by poorly represented cells in the biopsy, thus selecting for specific tumor molecular characteristics [45] All our HGSOC PDO lines exhibited a high sensitivity to THZ531 treatment, with half maximal inhibition of cell growth in the nanomolar range (70–200 nM). More importantly, our study indicates that THZ531 synergizes with drugs currently in use for this disease, such as Olaparib. The PDO lines that were thoroughly analyzed for combined treatments are wild type for *BRCA1/2* and resistant to Olaparib as single agent (IC_50_ ≥ 11µM). Nevertheless, combined treatment with THZ531 significantly enhanced the efficacy of the PARPi. Thus, inhibition of CDK12/13 activity can be exploited to rescue the sensitivity of BRCA1/2-proficient HGSOC to PARPi. Noteworthy, while the impact of CDK12 inhibition or deletion on the sensitivity to Olaparib in HGSOC cells was previously suggested, the results were discordant. Earlier studies indicated that ablation of CDK12 function, or introduction of tumor-related mutations, conferred sensitivity to PARPi [12, 15]. However, a recent genomic screen employing six ovarian cancer cell lines indicated that knockout of *CDK12* did not alter the response to Olaparib [46]. While this discrepancy may rely on the different cell lines used for these studies, it is also possible that CDK13 activity compensates for lack of CDK12 and affects sensitivity to PARPi in HGSOC cells. In support of this notion, transcriptome analysis of cells expressing analog-sensitive CDK12 and CDK13 indicated that their single inhibition exerted minimal effects on cell viability, whereas their concomitant inhibition potently induced gene expression dysregulation and cell death [17]. Moreover, these kinases were shown to cooperate also in ovarian cancer cells [16], further suggesting that dual CDK12/13 inhibition is required to block their oncogenic functions in HGSOC.

Our study also indicates that THZ531 enhances the cytotoxicity of Paclitaxel, a chemotherapeutic drug used as first line agent in HGSOC [1, 2]. As in the case of Olaparib, THZ531 synergized with Paclitaxel in all PDO lines tested, albeit with variable extent. Taxols prevent mitotic spindle dynamics by stabilizing microtubules, thus inhibiting cell division at the G2/M phase of the cell cycle [47]. We observed that treatment with THZ531 blocks cell cycle at the G2/M phase, with stalled cells displaying DNA lesions. Noteworthy, THZ531 altered the expression of several genes involved in mitotic spindle assembly (Fig. 4d), an effect which may underlie the G2/M block observed in OVCAR3 cells and the enhanced sensitivity of THZ531-treated HGSOC PDOs to Paclitaxel. On the other hand, the DNA breaks may be consequent to defects occurring during DNA duplication due to unresolved replication stress. Indeed, THZ531 impaired splicing and expression of ATRIP, a protein required for the recruitment of the ATR kinase at DNA lesions caused by replication stress. Accordingly, we observed synergism between THZ531 and inhibitors of this pathway. Thus, while most studies have linked CDK12 inhibition to higher sensitivity to PARPi, we now show that treatment of HGSOC cells with THZ531 also impairs the ATR signalling pathway. Moreover, we report that ATR inhibition sensitizes PARPi-resistant HGSOC cells and PDOs to Olaparib, a strategy that could be translated in the clinical setting. The effects of drugs targeting the ATR pathway were variable among the PDO lines. A significant synergism with the ATR inhibitor was observed only for PDO-41.3, whereas THZ531 synergized with CHK1 inhibition in PDO-24.3 and PDO41.3. Although these two drugs target the same signaling axis, our data suggest that individual tumors may be differentially sensitive to combined treatments. This is exemplified by PDO-24.3, which exhibited synergic response to THZ531 in combination with the CHK1 but not the ATR inhibitor.

Analysis of defective intron splicing caused by CDK12/13 inhibition highlighted new actionable targets for HGSOC. In this work, we focused on genes or pathways that are targetable by clinically approved drugs. For instance, inhibition of CDK12/13 impairs splicing of intron 2 in the *EGFR* gene. Treatment with THZ531 reduced the EGFR protein levels and sensitized HGSOC cells and PDOs to treatment with Lapatinib, a chemical inhibitor of the EGFR kinase activity currently in use for *HER2*-amplified breast cancer [48]. Thus, our study suggests that concomitant inhibition of EGFR expression (THZ531) and activity (Lapatinib) may improve the efficacy of therapeutic approaches for HGSOC. Likewise, we identified the *RPTOR* gene as a new target CDK12/13 activity. RPTOR is an adaptor protein required for the functionality of the mTORC1 complex, which drives protein synthesis and metabolic responses to external and internal cues [41]. The mTORC1 pathway is dysregulated in human cancers and inhibitors of this complex, such as RAD001 (Everolimus), are in clinical use for renal cancer and neuroendocrine tumors [49]. HGSOC cells and PDOs are relatively resistant to RAD001. However, treatment with THZ531 reduces the expression of the full-length RPTOR transcript, inhibits the mTORC1 pathway and sensitizes HGSOC cells to RAD001. Thus, our data suggest that CDK12/13 inhibition can generate vulnerabilities in HGSOC by specifically targeting the expression of genes involved in oncogenic pathways.

CDK12 and CDK13 have gained considerable attention as powerful oncogenic targets in the last years, especially for MYC-driven cancers [8, 9]. Herein, we report that more than 80% of HGSOC express functional CDK12 and/or CDK13 and that HGSOC cell models are highly sensitive to their pharmacologic inhibition. Unfortunately, approved drugs for these kinases are not available yet. Nevertheless, our findings suggest that preclinical studies using CDK12/13 inhibitors may translate in the clinical setting. Investigation of the transcriptome changes induced by CDK12/13 inhibition highlighted several oncogenic genes and pathways, whose concomitant impairment may underline the high efficacy of THZ531. In support of this hypothesis, we proved that combined inhibition of the mTORC1 complex and EGFR exerted a synergic effect on HGSOC cells and PDOs. Synergy was also observed between RAD001 and Trametininb, a clinically approved drug targeting downstream effector kinases of the EGFR pathway [44]. Trametinib represents a new standard-of-care option for women with recurrent low-grade serous ovarian carcinoma and displayed a good response also in a HGSOC patient that relapsed to previous treatments [50, 51]. Since EGFR and mTORC1 drive central mitogenic pathways in the cell and are frequently hyperactivated in human cancers, it is likely that their concomitant inhibition by THZ531 causes the strong repression of proliferation observed in our study. On the other hand, this evidence could support clinical studies testing the therapeutic efficacy of combined treatment with mTORC1 and EGFR inhibitors in HGSOC. Likewise, the synergism between Olaparib and Ceralasertib may suggest a strategy to treat HGSOC patients that do not harbor mutations in HR genes. More in general, mining of the RNA-seq data provided in our study may uncover other clinically relevant vulnerabilities for HGSOC. Further investigation of such potential vulnerabilities may allow to develop new second line targeted treatments for those patients who do not respond to standard chemotherapeutic regimens, or that relapse after initial response. Nevertheless, one limitation of our study is related to the genome-wide analysis that was performed in HGSOC cell line and not PDOs. Although we validated the results in PDOs for a limited number of genes, future transcriptomic analyses of primary samples are required to confirm the widespread effect of CDK12/13 inhibition on intron retention and gene inactivation in HGSOC.

## Conclusion

In this study, we provide evidence that inhibition of CDK12/13 enhances the sensitivity of HGSOC primary cells to agents already in use for this disease (i.e. Paclitaxel and Olaparib). Moreover, transcriptomic analyses of the effects of CDK12/13 inhibition highlighted new potential vulnerabilities of HGSOC to drugs that are clinically approved for other cancer types. Thus, our study confirms that PDOs represent a valuable platform for understanding of HGSOC biology and for screening known and new potential drugs. Although these findings need to be supported by further experimental evidence, they pave the ground for a dynamic ex-vivo approach aimed at uncovering actionable vulnerabilities of HGSOC.

## Supplementary Information

**Additional file 1: Table S1.** Primers for semi-quantitative and real time PCR.

**Additional file 2: Table S2.** Clinicopathological features of HGSOC patients and their corresponding organoids.

**Additional file 3. **RNA-seq datasets: regulated genes and pattern exons.

**Additional file 4: Figure S1.** CDK12/13 alterations in HGSOC. **Figure S2.** CDK12/CDK3 inhibition impairs OVCAR3 cell growth. **Figure S3.** Trascriptomic analysis of the effect of THZ531 in OVCAR3 cells. **Figure S4.** CDK12/13 inhibition impairs splicing and expression of cancer-relevant genes in HGSOC. **Figure S5.** Transcriptome analysis of OVCAR8 cells upon inhibition of CDK12/13. **Figure S6.** THZ531 treatment in OVCAR3 cells overcomes resistance to standard chemotherapeutic treatments. **Figure S7.** Cytotoxicity effect of clinically relevant inhibitors on HGSOC PDO lines.

## Data Availability

All data generated during this study are included in this published article [and its supplementary information files]. During the current study, the datasets analysed are publicly available in BioPortal repository (https://www.cbioportal.org/).

## Abbreviations

ALE: Alternative last exons
ATR: Ataxia Telangiectasia and Rad3 Related
ATRIP: ATR Interacting Protein
BME: Basement Membrane Extract
BRCA WT: BReast CAncer gene wilt type
BRCA: BReast CAncer gene 2
BRCA1: BReast CAncer gene 1
BrdU: Bromodeoxyuridine
CDK12: Cyclin dependent kinases 12
CDK13: Cyclin dependent kinases 13
CHK1: Checkpoint kinase 1
CI: Combination Index
CK20: Cytokeratin 20
CK7: Cytokeratin 7
Css/Cmax: Concentration steady state/maximum concentration
CTD: Carboxyl-terminal domain
DAB: 3,3′-Diaminobenzidine substrate;
DDR: DNA damage response
DMEM: Dulbeccoʼs modifed Eagle′s medium
DNA: Deoxyribonucleic acid
EGFR: Epidermal growth factor receptor
GE: Gene expression
HE: Haematoxylin and Eosin
HGSOC: High grade serous ovarian cancer
IC50: Half maximal inhibitory concentration
IR: Intron retention
KEGG: Kyoto Encyclopedia of Genes and Genomes
OS: Overall survival
OV.GEM: Anonymous numerical codes for primary cell lines
PARP: Poly (ADP-ribose) polymerase
PAX8: Paired box gene 8
PBS: Phosphate bufered saline
PDO: Patient-derived organoids
POLH: DNA Polymerase Eta
RBM47: RNA Binding Motif Protein 47
RNA: Ribonucleic acid
RNAPII: RNA polymerase II
RNA-seq: RNA sequencing
RP: RNA processing
RPTOR: Regulatory Associated Protein of MTOR Complex 1
RT-qPCR: Quantitative real-time Polymerase chain reaction
TGCA: The Cancer Genome Atlas
VEGF: Vascular endothelial growth factor
WT1: Wilms Tumor 1

## References

[1] Lheureux S, Gourley C, Vergote I, Oza AM (2019). Epithelial ovarian cancer. Lancet.

[2] Lorusso D, Ceni V, Daniele G, Salutari V, Pietragalla A, Muratore M (2020). Newly diagnosed ovarian cancer: Which first-line treatment?. Cancer Treat Rev.

[3] Bowtell DD, Böhm S, Ahmed AA, Aspuria PJ, Bast RC, Beral V (2015). Rethinking ovarian cancer II: reducing mortality from high-grade serous ovarian cancer. Nat Rev Cancer.

[4] Cancer Genome Atlas Research Network (2011). Integrated genomic analyses of ovarian carcinoma. Nature.

[5] Levine AJ (2020). p53: 800 million years of evolution and 40 years of discovery. Nat Rev Cancer.

[6] Curtin NJ, Szabo C (2020). Poly(ADP-ribose) polymerase inhibition: past, present and future. Nat Rev Drug Discov.

[7] George A, Kaye S, Banerjee S (2017). Delivering widespread BRCA testing and PARP inhibition to patients with ovarian cancer. Nat Rev Clin Oncol.

[8] Chou J, Quigley DA, Robinson TM, Feng FY, Ashworth A (2020). Transcription-Associated Cyclin-Dependent Kinases as Targets and Biomarkers for Cancer Therapy. Cancer Discov.

[9] Naro C, Bielli P, Sette C (2021). Oncogenic dysregulation of pre-mRNA processing by protein kinases: challenges and therapeutic opportunities. FEBS J.

[10] Pilarova K, Herudek J, Blazek D (2020). CDK12: cellular functions and therapeutic potential of versatile player in cancer. NAR Cancer..

[11] Blazek D, Kohoutek J, Bartholomeeusen K, Johansen E, Hulinkova P, Luo Z (2011). The Cyclin K/Cdk12 complex maintains genomic stability via regulation of expression of DNA damage response genes. Genes Dev.

[12] Bajrami I, Frankum JR, Konde A, Miller RE, Rehman FL, Brough R (2014). Genome-wide profiling of genetic synthetic lethality identifies CDK12 as a novel determinant of PARP1/2 inhibitor sensitivity. Cancer Res.

[13] Johnson SF, Cruz C, Greifenberg AK, Dust S, Stover DG, Chi D (2016). CDK12 Inhibition Reverses De Novo and Acquired PARP Inhibitor Resistance in BRCA Wild-Type and Mutated Models of Triple-Negative Breast Cancer. Cell Rep.

[14] Ekumi KM, Paculova H, Lenasi T, Pospichalova V, Bösken CA, Rybarikova J (2015). Ovarian carcinoma CDK12 mutations misregulate expression of DNA repair genes via deficient formation and function of the Cdk12/CycK complex. Nucleic Acids Res.

[15] Joshi PM, Sutor SL, Huntoon CJ, Karnitz LM (2014). Ovarian cancer-associated mutations disable catalytic activity of CDK12, a kinase that promotes homologous recombination repair and resistance to cisplatin and poly(ADP-ribose) polymerase inhibitors. J Biol Chem.

[16] Cheng L, Zhou S, Zhou S, Shi K, Cheng Y, Cai MC (2022). Dual inhibition of CDK12/CDK13 targets both tumor and immune cells in ovarian cancer. Cancer Res.

[17] Fan Z, Devlin JR, Hogg SJ, Doyle MA, Harrison PF, Todorovski I (2020). CDK13 cooperates with CDK12 to control global RNA polymerase II processivity. Sci Adv..

[18] Dubbury SJ, Boutz PL, Sharp PA (2018). CDK12 regulates DNA repair genes by suppressing intronic polyadenylation. Nature.

[19] Krajewska M, Dries R, Grassetti AV, Dust S, Gao Y, Huang H (2019). CDK12 loss in cancer cells affects DNA damage response genes through premature cleavage and polyadenylation. Nat Commun.

[20] Buttarelli M, Ciucci A, Palluzzi F, Raspaglio G, Marchetti C, Perrone E (2022). Identification of a novel gene signature predicting response to first-line chemotherapy in BRCA wild-type high-grade serous ovarian cancer patients. J Exp Clin Cancer Res.

[21] Harris PA, Taylor R, Thielke R, Payne J, Gonzalez N, Conde JG (2009). Research electronic data capture (REDCap)—a metadata-driven methodology and workflow process for providing translational research informatics support. J Biomed Inform.

[22] Chou TC, Talalay P (1984). Quantitative analysis of dose-effect relationships: the combined effects of multiple drugs or enzyme inhibitors. Adv Enzyme Regul.

[23] Naro C, Jolly A, Di Persio S, Bielli P, Setterblad N, Alberdi AJ (2017). An Orchestrated Intron Retention Program in Meiosis Controls Timely Usage of Transcripts during Germ Cell Differentiation. Dev Cell.

[24] Naro C, Pellegrini L, Jolly A, Farini D, Cesari E, Bielli P (2019). Functional Interaction between U1snRNP and Sam68 Insures Proper 3' End Pre-mRNA Processing during Germ Cell Differentiation. Cell Rep.

[25] Naro C, De Musso M, Delle Monache F, Panzeri V, de la Grange P, Sette C (2021). The oncogenic kinase NEK2 regulates an RBFOX2-dependent pro-mesenchymal splicing program in triple-negative breast cancer cells. J Exp Clin Cancer Res.

[26] Zeng M, Kwiatkowski NP, Zhang T, Nabet B, Xu M, Liang Y (2018). Targeting MYC dependency in ovarian cancer through inhibition of CDK7 and CDK12/13. Elife.

[27] Zhang T, Kwiatkowski N, Olson CM, Dixon-Clarke SE, Abraham BJ, Greifenberg AK (2016). Covalent targeting of remote cysteine residues to develop CDK12 and CDK13 inhibitors. Nat Chem Biol.

[28] Quereda V, Bayle S, Vena F, Frydman SM, Monastyrskyi A, Roush WR, Duckett DR (2019). Therapeutic Targeting of CDK12/CDK13 in Triple-Negative Breast Cancer. Cancer Cell.

[29] Drost J, Clevers H (2018). Organoids in cancer research. Nat Rev Cancer.

[30] Tuveson D, Clevers H (2019). Cancer modeling meets human organoid technology. Science.

[31] Hill SJ, Decker B, Roberts EA, Horowitz NS, Muto MG, Worley MJ (2018). Prediction of DNA Repair Inhibitor Response in Short-Term Patient-Derived Ovarian Cancer Organoids. Cancer Discov.

[32] Kopper O, de Witte CJ, Lõhmussaar K, Valle-Inclan JE, Hami N, Kester L (2019). An organoid platform for ovarian cancer captures intra- and interpatient heterogeneity. Nat Med.

[33] Nero C, Vizzielli G, Lorusso D, Cesari E, Daniele G, Loverro M (2021). Patient-derived organoids and high grade serous ovarian cancer: from disease modeling to personalized medicine. J Exp Clin Cancer Res.

[34] de Witte CJ, Espejo Valle-Inclan J, Hami N, Lõhmussaar K, Kopper O, Vreuls CPH (2020). Patient-derived ovarian Cancer Organoids mimic clinical response and exhibit heterogeneous inter- and Intrapatient drug responses. Cell Rep.

[35] Hanahan D, Weinberg RA (2011). Hallmarks of cancer: the next generation. Cell.

[36] Harlen KM, Churchman LS (2017). The code and beyond: transcription regulation by the RNA polymerase II carboxy-terminal domain. Nat Rev Mol Cell Biol.

[37] Panzeri Panzeri V, Pieraccioli M, Cesari E, de la Grange P, Sette C. CDK12/13 promote splicing of proximal introns by enhancing the interaction between RNA polymerase II and the splicing factor SF3B1. Nucleic Acids Res. 2023:gkad258. 10.1093/nar/gkad258. Online ahead of print. 10.1093/nar/gkad258 PMC10287901 37026485

[38] Tian B, Manley JL (2017). Alternative polyadenylation of mRNA precursors. Nat Rev Mol Cell Biol.

[39] Mhawech-Fauceglia P, Wang D, Samrao D, Godoy H, Ough F, Liu S (2012). Pair Box 8 (PAX8) protein expression in high grade, late stage (stages III and IV) ovarian serous carcinoma. Gynecol Oncol.

[40] Lin L, Shi K, Zhou S, Cai MC, Zhang C, Sun Y (2022). SOX17 and PAX8 constitute an actionable lineage-survival transcriptional complex in ovarian cancer. Oncogene.

[41] Dibble CC, Cantley LC (2015). Regulation of mTORC1 by PI3K signaling. Trends Cell Biol.

[42] Parashar D, Nair B, Geethadevi A, George J, Nair A, Tsaih SW (2020). Peritoneal Spread of Ovarian Cancer Harbors Therapeutic Vulnerabilities Regulated by FOXM1 and EGFR/ERBB2 Signaling. Cancer Res.

[43] Nam EA, Cortez D (2011). ATR signalling: more than meeting at the fork. Biochem J.

[44] Zeiser R (2014). Trametinib. Recent Results Cancer Res.

[45] Kinker GS, Greenwald AC, Tal R, Orlova Z, Cuoco MS, McFarland JM (2020). Pan-cancer single-cell RNA-seq identifies recurring programs of cellular heterogeneity. Nat Genet.

[46] Coelho R, Tozzi A, Disler M, Lombardo F, Fedier A, López MN (2022). Overlapping gene dependencies for PARP inhibitors and carboplatin response identified by functional CRISPR-Cas9 screening in ovarian cancer. Cell Death Dis.

[47] Schiff PB, Horwitz SB (1980). Taxol stabilizes microtubules in mouse fibroblast cells. Proc Natl Acad Sci USA.

[48] Bundred N, Porta N, Brunt AM, Cramer A, Hanby A, Shaaban AM (2022). Combined Perioperative Lapatinib and Trastuzumab in Early HER2-Positive Breast Cancer Identifies Early Responders: Randomized UK EPHOS-B Trial Long-Term Results. Clin Cancer Res.

[49] Hasskarl J (2018). Everolimus. Recent Results Cancer Res.

[50] Gershenson DM, Miller A, Brady WE, Paul J, Carty K, Rodgers W (2022). Trametinib versus standard of care in patients with recurrent low-grade serous ovarian cancer (GOG 281/LOGS): an international, randomised, open-label, multicentre, phase 2/3 trial. Lancet.

[51] Cappuccio S, Distefano MG, Ghizzoni V, Fagotti A, Scambia G (2020). Trametinib response in heavily pretreated high-grade ovarian cancer: One step towards precision medicine. Gynecol Oncol Rep.

