# Supplementary figures and images for: Dual inhibition of CDK12 and CDK13 uncovers actionable vulnerabilities in patient-derived ovarian cancer organoids
Source: J Exp Clin Cancer Res. 2023 May 18;42:126. doi: 10.1186/s13046-023-02682-5 (PMC10193743; doi:10.1186/s13046-023-02682-5)

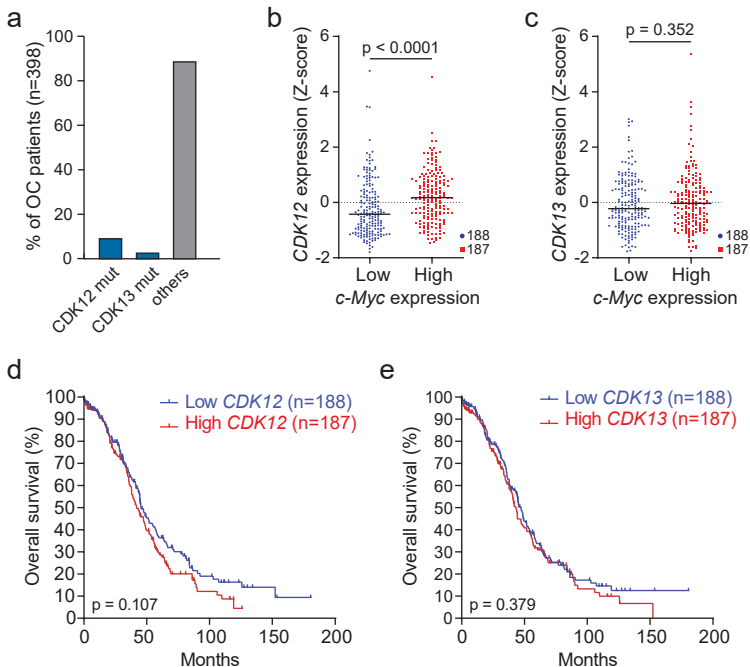

Figure S1

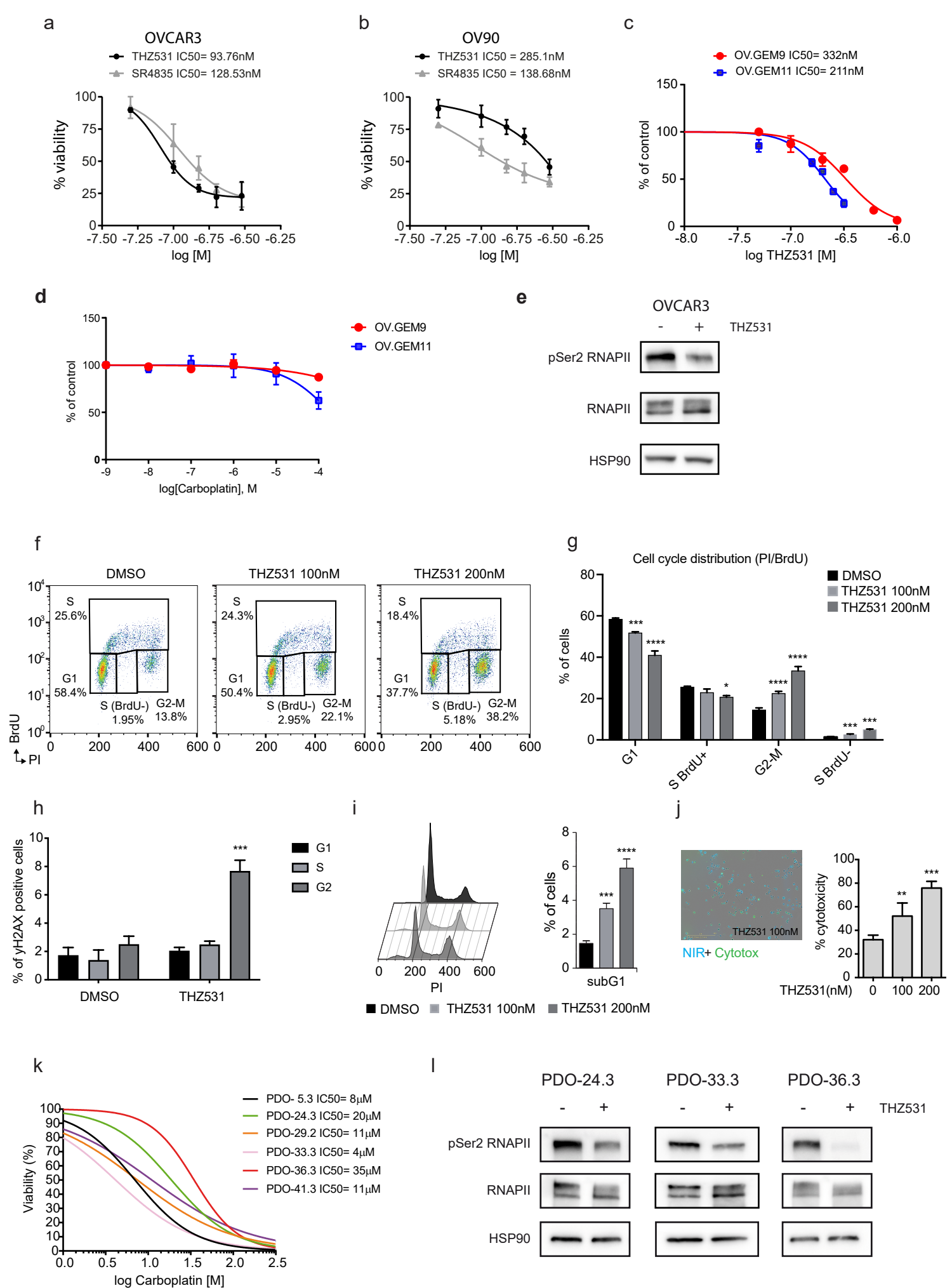

Figure S2

a

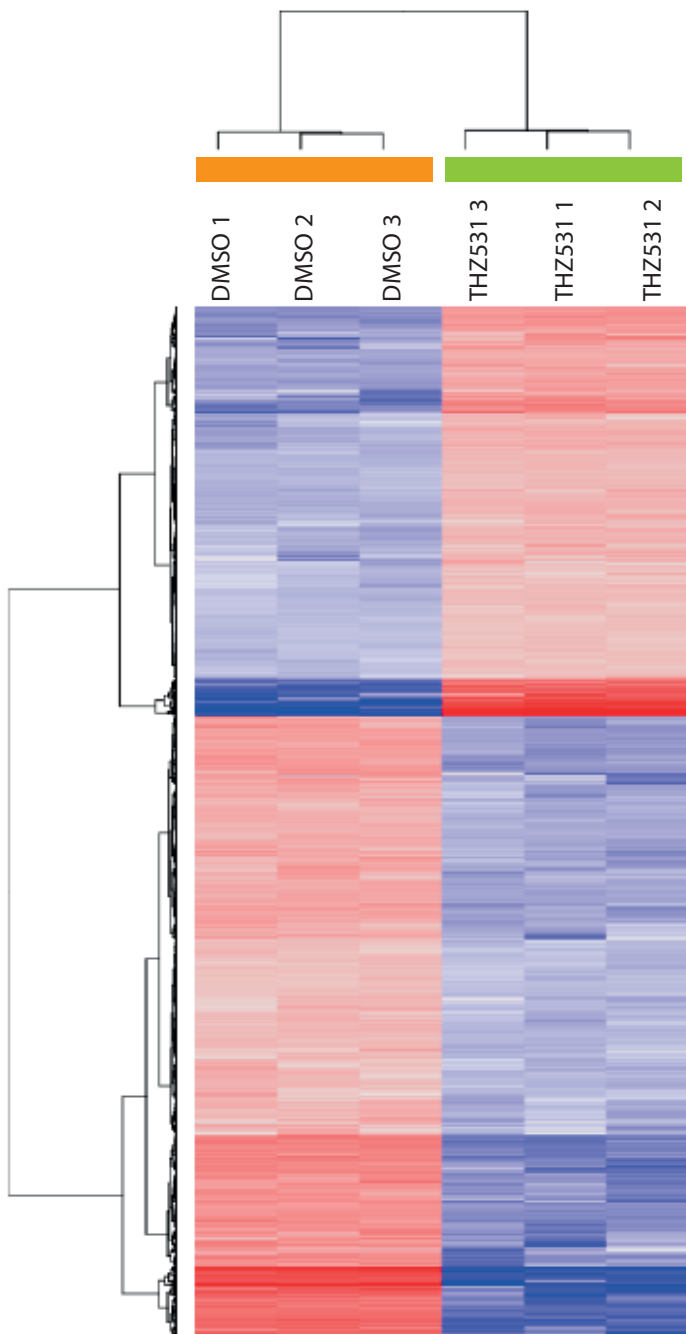

b

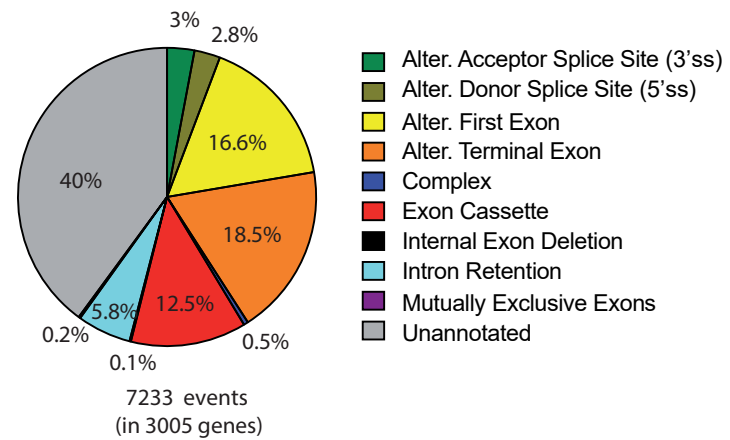

c

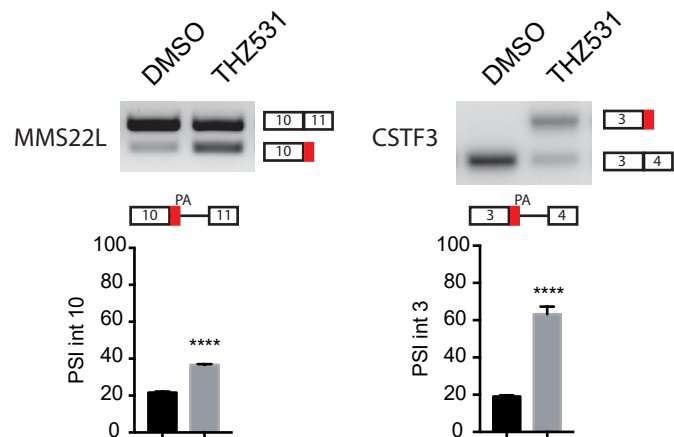

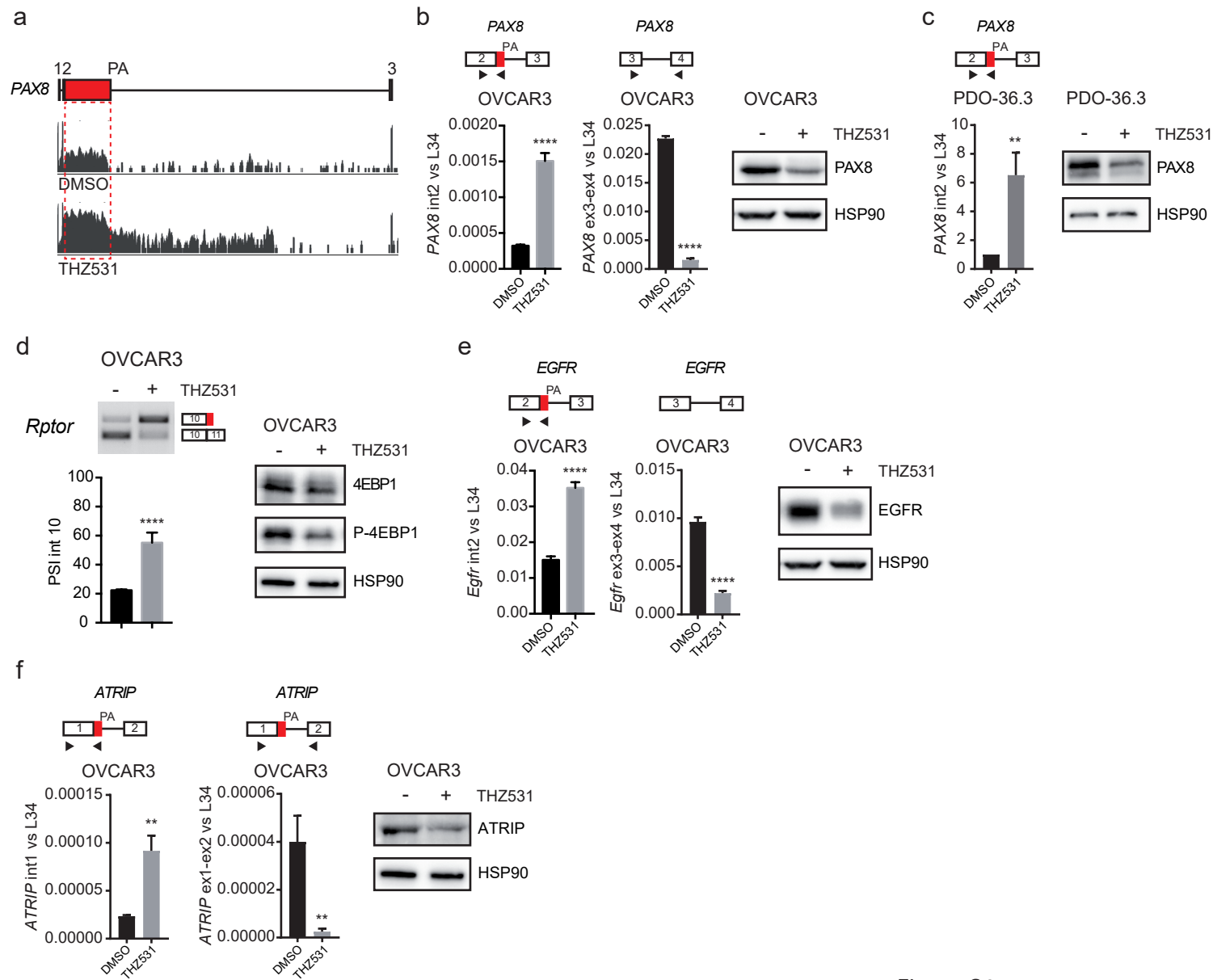

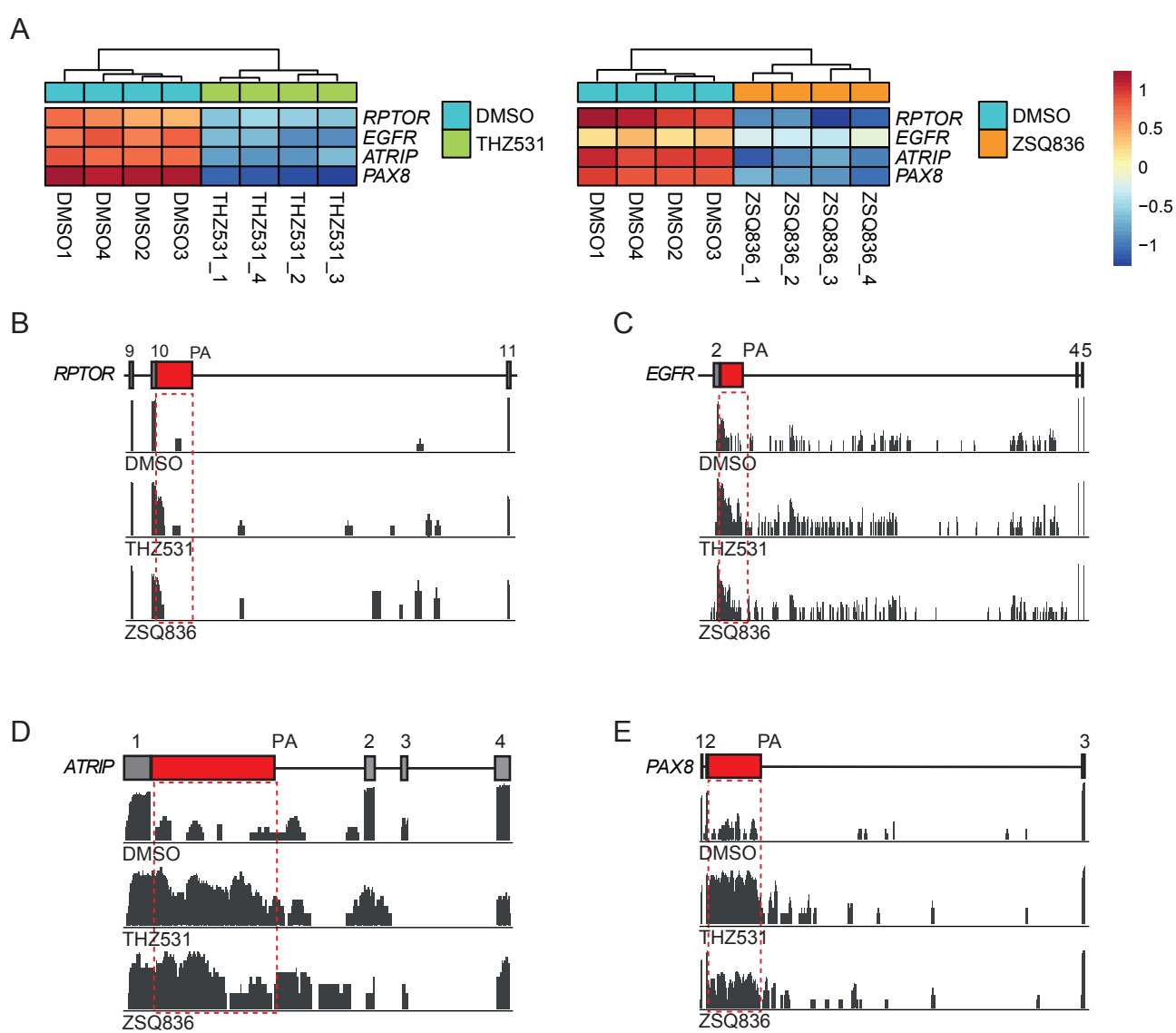

Figure S5

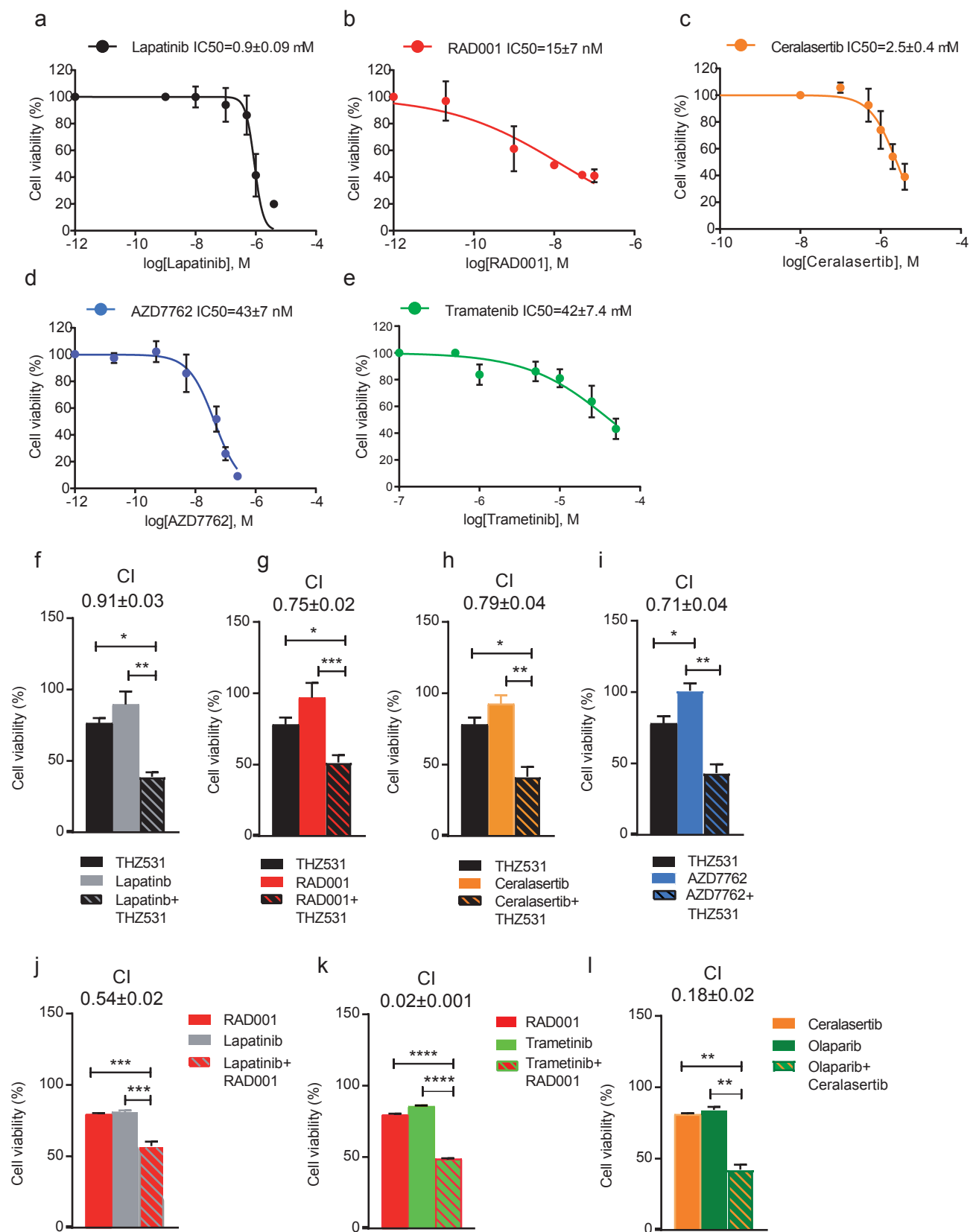

Figure S6

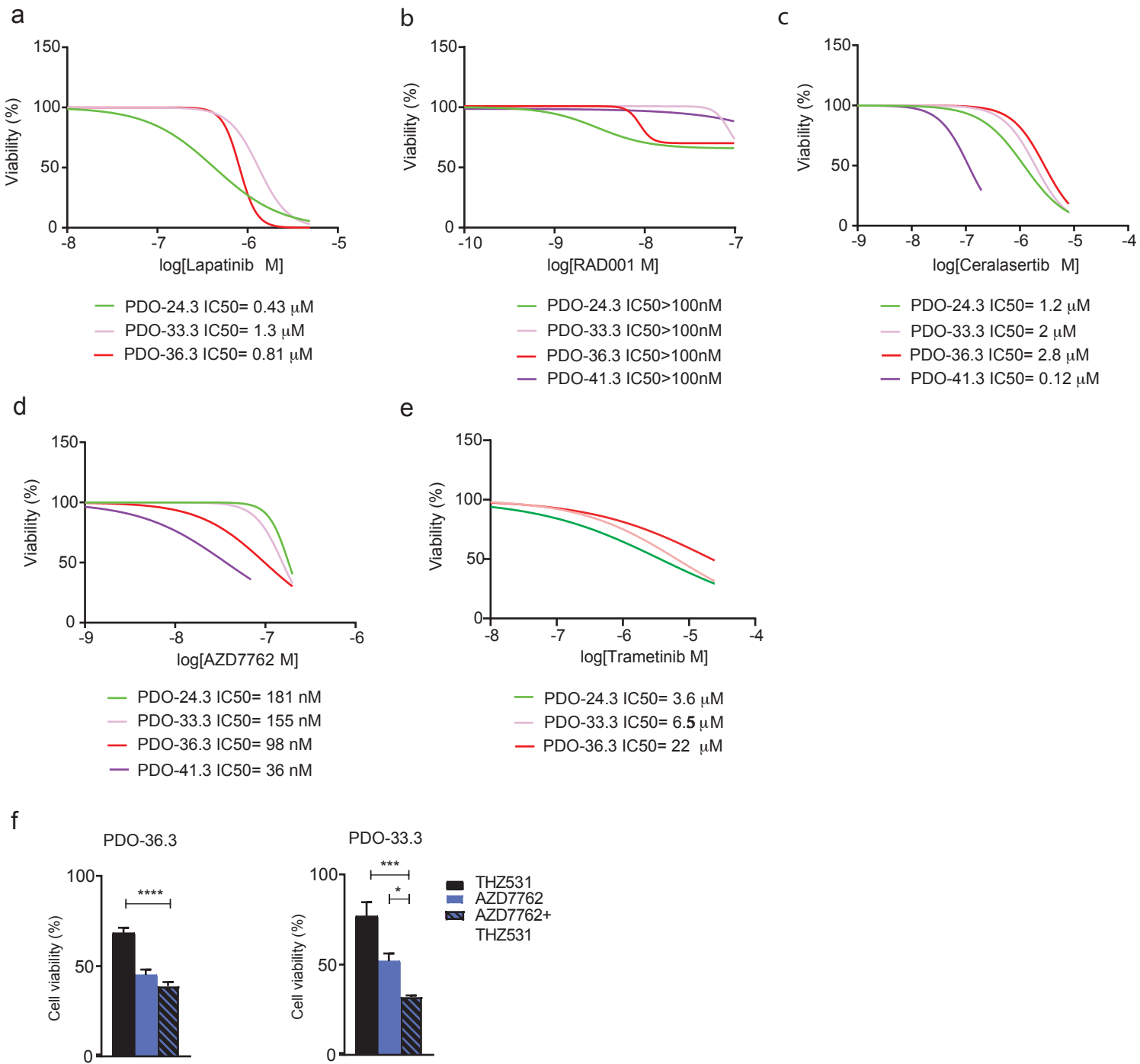

Figure S7

Supplement: Supplementary file 4 — Additional file 4: Figure S1. CDK12/13 alterations in HGSOC. Figure S2. CDK12/CDK3 inhibition impairs OVCAR3 cell growth. Figure S3. Trascriptomic analysis of the effect of THZ531 in OVCAR3 cells. Figure S4. CDK12/13 inhibition impairs splicing and expression of cancer-relevant genes in HGSOC. Figure S5. Transcriptome analysis of OVCAR8 cells upon inhibition of CDK12/13. Figure S6. THZ531 treatment in OVCAR3 cells overcomes resistance to standard chemotherapeutic treatments. Figure S7. Cytotoxicity effect of clinically relevant inhibitors on HGSOC PDO lines. [file 13046_2023_2682_MOESM4_ESM.pdf]
